# Inert Liquid Exfoliation and Langmuir-Type Thin Film Deposition of Semimetallic Metal Diborides

**DOI:** 10.1021/acsnano.4c04626

**Published:** 2024-10-09

**Authors:** Kevin Synnatschke, Alina Müller, Cian Gabbett, Michael Johannes Mohn, Adam G. Kelly, Kseniia Mosina, Bing Wu, Eoin Caffrey, Oran Cassidy, Claudia Backes, Zdenek Sofer, Ute Kaiser, Jonathan N. Coleman

**Affiliations:** † School of Physics, CRANN & AMBER Research Centres, Trinity College Dublin, Dublin 2, Ireland; ‡ Chair of Applied Physical Chemistry, Heidelberg University, Im Neuenheimer Feld 253, 69120 Heidelberg, Germany; § Central Facility of Electron Microscopy, Electron Microscopy Group of Materials Science, Ulm University, Albert-Einstein-Allee 11, 89081 Ulm, Germany; ∥ Department of Inorganic Chemistry, Faculty of Chemical Technology, University of Chemistry and Technology Prague, Technická 5, Prague16628 Czech Republic; ⊥ Institute of Physical Chemistry, University of Kassel, Heinrich-Plett-Straße 40, 34132 Kassel, Germany

**Keywords:** liquid/liquid interface deposition, morphology control, thin films, 2D nanomaterials, inert exfoliation, liquid-phase exfoliation, nanomaterial stability

## Abstract

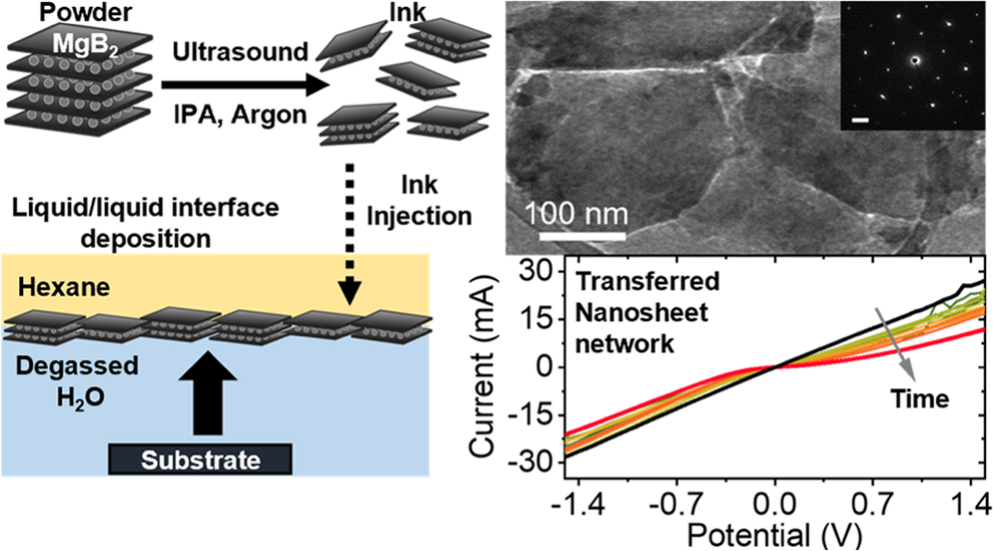

Graphite is one of only a few layered materials that can be exfoliated into nanosheets with semimetallic properties, which limits the applications of nanosheet-based electrodes to material combinations compatible with the work function of graphene. It is therefore important to identify additional metallic or semimetallic two-dimensional (2D) nanomaterials that can be processed in solution for scalable fabrication of printed electronic devices. Metal diborides represent a family of layered non-van der Waals crystals with semimetallic properties for all nanosheet thicknesses. While previous reports show that the exfoliated nanomaterial is prone to oxidation, we demonstrate a readily accessible inert exfoliation process to produce quasi-2D nanoplatelets with intrinsic material properties. For this purpose, we demonstrate the exfoliation of three representative metal diborides (MgB_2_, CrB_2_, and ZrB_2_) under inert conditions. Nanomaterial is characterized using a combination of transmission electron microscopy, scanning electron microscopy, atomic force microscopy, IR, and UV–vis measurements, with only minimal oxidation indicated postprocessing. By depositing the pristine metal diboride nanoplatelets as thin films using a Langmuir-type deposition technique, the ohmic behavior of the networks is validated. Furthermore, the material decomposition is studied by using a combination of electrical and optical measurements after controlled exposure to ambient conditions. Finally, we report an efficient, low-cost approach for sample encapsulation to protect the nanomaterials from oxidation. This is used to demonstrate low-gauge factor strain sensors, confirming metal diboride nanosheets as a suitable alternative to graphene for electrode materials in printed electronics.

## Introduction

In recent years, liquid exfoliated two-dimensional (2D) materials have become important for a range of applications in areas from printed electronics^1−4^ to sensing^5−8^ to energy storage.^9−12^ In the former area, many researchers have commented that the family of 2D materials is particularly important as it includes conducting, semiconducting, and insulating members^3,13^ and so can contribute all of the building blocks of electronic devices. Indeed, various researchers have reported all-2D printed electronic devices where electrodes and dielectric and channel materials are fabricated from different 2D materials.^1,3^ However, while there are many semiconducting 2D materials that can be liquid exfoliated and solution processed, there are significantly fewer insulating and conducting ones. In the latter category, the main materials are graphene,^14^ MXenes,^15^ conducting metal chalcogenides (e.g., 1T MoS_2_),^16,17^ and silver nanoplatelets.^18^ Of these, the most common material, graphene, yields solution-processed films, which are not particularly conductive, generally 10^3^–10^5^ S/m.^19−23^ Thus, it would be advantageous to increase the number of solution processable, conducting 2D materials.

**Figure 1:**
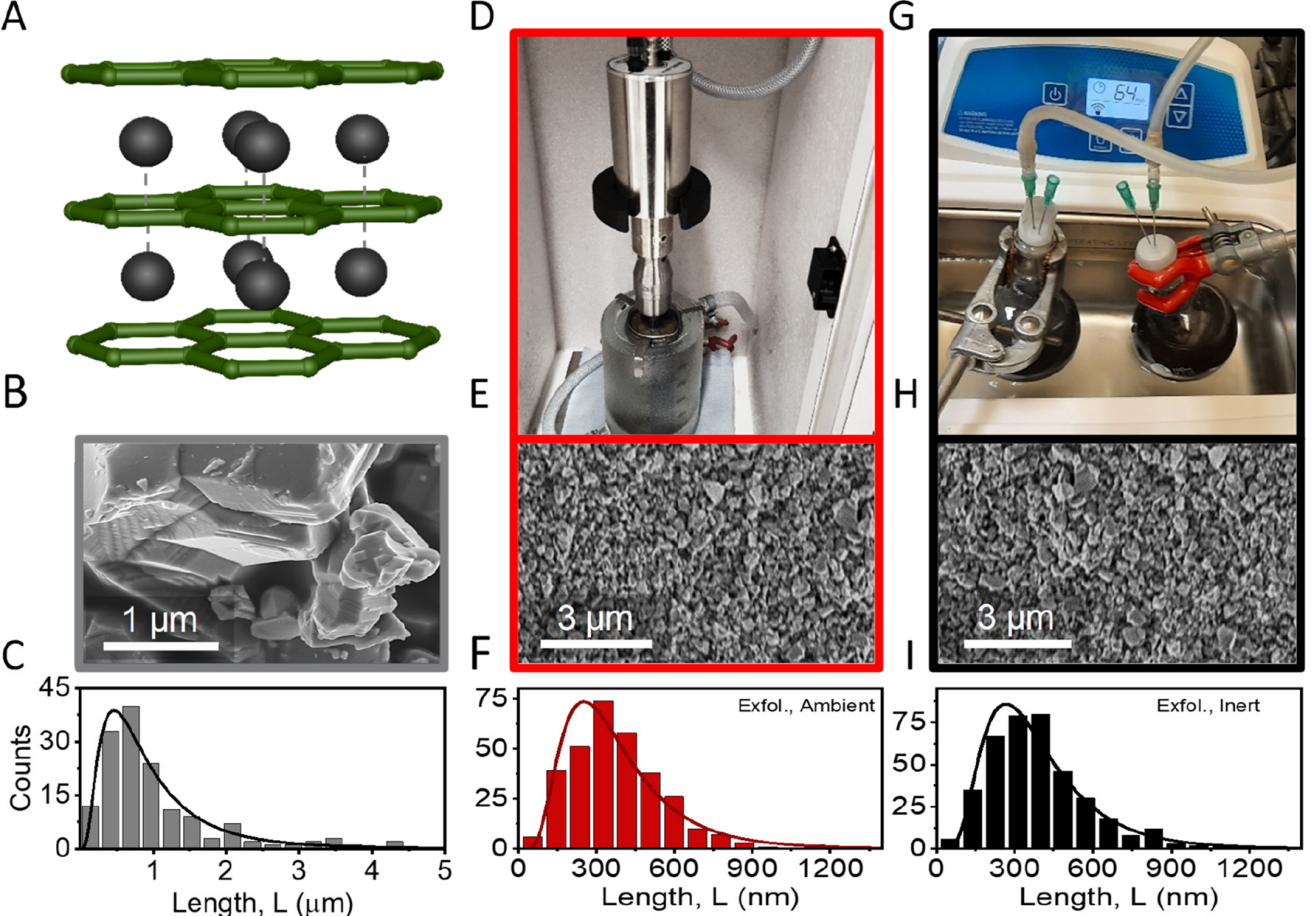
SEM comparison of metal diboride nanoplatelets made under ambient and inert conditions. (A) Hexagonal AlB_2_-type crystal structure, typical for layered metal diborides such as MgB_2_. Layers of negatively charged boron sheets (represented by a green hexagonal lattice) are sandwiched between Mg^2+^ ions (black spheres and square lattice). (B, C) Representative SEM image (B) and size distribution of MgB_2_ crystallites (C) in the powder used as a starting material. (D) Photograph of the setup used for exfoliation under ambient conditions. (E, F) Scanning electron microscopy (SEM) image showing an overview of drop cast nanosheets after exfoliation (E) using the setup shown in panel D and the corresponding size distribution of MgB_2_ nanosheets determined from SEM imaging (F). (G) Photograph of the setup used for exfoliation under inert conditions. (H, I) SEM image showing an overview of drop cast nanosheets after exfoliation (H) using the setup shown in panel G and corresponding size distribution of MgB_2_ nanosheets determined from SEM imaging (I).

Layered metal diborides form a family of layered non-van der Waals semimetallic ceramics composed of bivalent metal ions sandwiched by negatively charged boron sheets with a general stoichiometry of MB_2_ (M = Al^2+^, Mg^2+^, Cr^2+^, Hf^2+^, Nb^2+^, Ta^2+^, Ti^2+^, V^2+^, Zr^2+^). All layered metal diborides crystallize in a hexagonal structure (space group *P*6/*mmm*), as schematically shown in Figure 1A.^24,25^ The semimetallic behavior of many members of this family results from dispersive nature of the energy bands over the whole Brillouin zone, with only few orbitals crossing the Fermi level.^26,27^ At room temperature, the resistivity of polycrystalline bulk MgB_2_ is ρ ∼ (1–10) × 10^–7^ Ω m (σ ∼ (0.1–1) × 10^7^ S/m) and dρ/d*T* > 0, consistent with metal-like behavior.^28,29^ We note that MgB_2_ undergoes a transition to superconducting behavior at 39 K.^29,30^

The electronic structure of MgB_2_ is intriguing due to its similarity to graphite. It features three bonding σ-bands from sp^2^ hybridization within the boron layer and a pair of π-bands from p_*z*_-orbital hybridization. The strong in-plane electron dispersion arises from the efficient p-orbital overlap between neighboring boron atoms, while interlayer overlap (especially p_*xy*_-orbitals) results in a small k_*z*_-dispersion (<1 eV). This leads to two cylindrical sheets around the Γ-A-line on the Fermi surface. The distinctive covalent character of unoccupied Γ states with p_*xy*_ symmetry crossing the Fermi level has been studied in theory and experimentally using charge density experiments, revealing its impact on transport characteristics and superconductivity while also indicating structural instabilities.^26,27^

In concise terms, this implies that the bonding nature between boron atoms in a metal diboride is considered covalent, while the interlayer bonding with ionized magnesium atoms possesses metallic attributes with delocalized electrons in the interstitial spaces.^27^ This stands in contrast to graphite, where covalent bonding within the carbon lattice is saturated, and the “cylindrical” sheets in the Fermi surface originate from π-bands with more two-dimensional character compared to the bands formed in metal diborides.^31^ This distinction holds significance as it results in interactions that are strong in the in-plane directions and reasonably strong in the out-of-plane direction for metal diborides. In contrast, the out-of-plane attraction in graphite relies solely on van der Waals interactions, leading to a significant anisotropy in the binding strength between the in-plane and out-of-plane directions. This distinction facilitates efficient exfoliation of graphite into reasonably high aspect ratio 2D nanosheets.^32^ The binding strength anisotropy is expected to be considerably lower for metal diborides.

Upon exfoliation, a less pronounced anisotropy of the binding energy is expected to result in nanosheets with a lower length/thickness aspect ratio.^32^ However, despite some reports on their sonication-assisted exfoliation in liquid media,^25,33−39^ only little quantitative information can be found in the literature on the attainable nanosheet dimensions of liquid exfoliated metal diborides, or how the exfoliation influences their electronic properties. Furthermore, prior studies suggest that the nanomaterial is susceptible to surface functionalization by oxide and hydroxide species when exposed to ambient or aqueous environments (see SI, Section 7 for an additional discussion and a comparison of results published in the literature).^25,33−42^

In this study, we provide the first quantitative insights into the size, electronic properties, and reactivity of pristine sonication-assisted liquid-phase exfoliated metal diborides. MgB_2_ powder is exfoliated in dry and degassed isopropanol, and the nanomaterials’ sensitivity to ambient oxygen is studied by comparing exfoliation under ambient and inert conditions. To remove unexfoliated material as well as oxidized material and impurities, nanosheet dispersions undergo two centrifugation steps, as described in more detail in the Methods/Experimental Section. Statistical analysis of nanoplatelet size and thickness is conducted through SEM and atomic force microscopy (AFM) measurements. Characterization includes UV–vis measurements, EDX spectroscopy, and IR transmittance, indicating significant oxidation for ambient-exfoliated samples. Nanoplatelets produced under inert conditions are further analyzed via transmission electron microscopy (TEM), confirming crystallinity. We further employ an inert Langmuir-type deposition for fabrication of thin films with high metallic conductivity. Nanosheet films and inks exposed to ambient conditions show material decomposition over time, demonstrated via decay of optical extinction and electrical conductivity. Half-lives for decomposition provide an upper limit for processing time, while encapsulation can be used to stabilize the films.

## Results/Discussion

### Assessing the Impact of Ambient Oxygen during Exfoliation

Prior to exfoliation, statistical SEM measurements were used to find the distribution of crystallite sizes found in the powdered starting material. These measurements confirm the layered character of the material (Figure 1B) and reveal a broad distribution of crystallite sizes with an average lateral size of ∼1 μm (Figure 1C).

**Figure 2:**
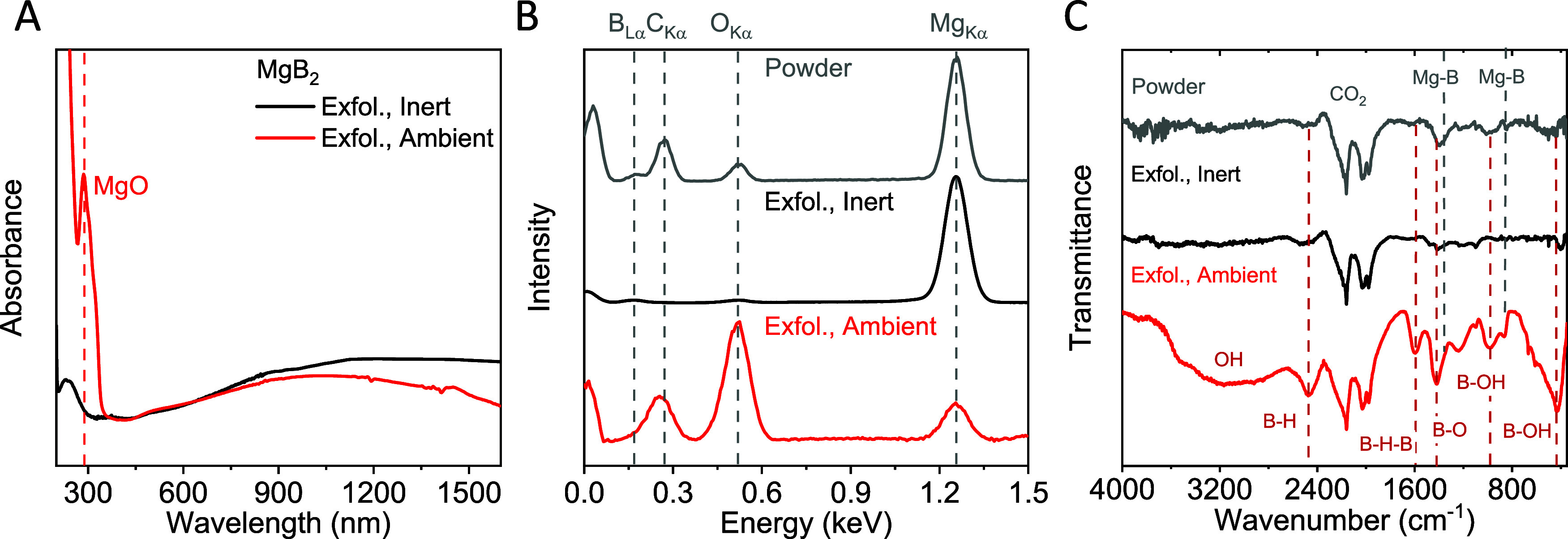
Spectroscopic analysis of MgB_2_ nanoplatelets prepared under inert conditions. (A) Absorbance measurements on nanosheet dispersions made under ambient (red) and inert conditions (black). An additional peak associated with MgO is found for the ambient sample. (B, C) EDX (B) and IR-transmittance (C) measurements for the starting material (gray) and nanosheets after exfoliation under ambient (red) and inert (black) conditions. The measurements suggest that both the starting and ambient-exfoliated materials contain oxide species, which is not evident in the inert exfoliated sample.

For material exfoliation, two samples of magnesium diboride nanosheets were prepared by sonication-assisted exfoliation in IPA for 7 h under ambient and inert conditions, respectively. Both dispersions were subjected to two centrifugation steps. The first at 200*g* is to remove unexfoliated material from the dispersion, and the second step at 3000*g* is to collect the nanosheets as sediment, leaving any small particles, soluble oxides, or impurities in the supernatant. Collecting the nanosheets as sediment allows one to redisperse the nanomaterial in small volumes of fresh solvent at the concentration of choice. All analysis was performed on samples prepared according to this procedure. The resulting inks from both ambient and inert exfoliation conditions were subjected to the same statistical microscopic analysis as the powder (Figure 1D–I). The SEM analysis shows no difference between the nanosheet sizes for the two samples prepared under ambient (Figure 1D–F) and inert (Figure 1G–I) conditions. In both cases, an average lateral nanosheet size of ∼350 nm is found by measuring the lateral size of >300 individual nanosheets for both samples. For further characterization, optical absorbance measurements on both dispersions have been carried out (Figure 2A). While both samples show a broad absorption feature over the entire accessible spectroscopic range, the sample exfoliated in ambient condition shows an additional peak in the UV region, which can be attributed to contributions from magnesium oxide.^43^ No such feature is observed for the inert sample, which shows only a weak peak at ∼260 nm. We attribute this feature to an optical excitation in pristine MgB_2_, which is masked by oxide contributions in the ambient sample. In addition, EDX and IR-transmittance measurements were performed on both samples, including the starting material as a reference (Figure 2B,C). Both measurements indicate that some oxides are present in the starting material. While no oxide contribution is observed in the spectra for nanosheets exfoliated under inert conditions, significant oxidation is evident in absorbance spectroscopy, EDX, and IR transmission after exfoliation in ambient conditions.

### Quantitative Analysis of the Nanomaterial

**Figure 3:**
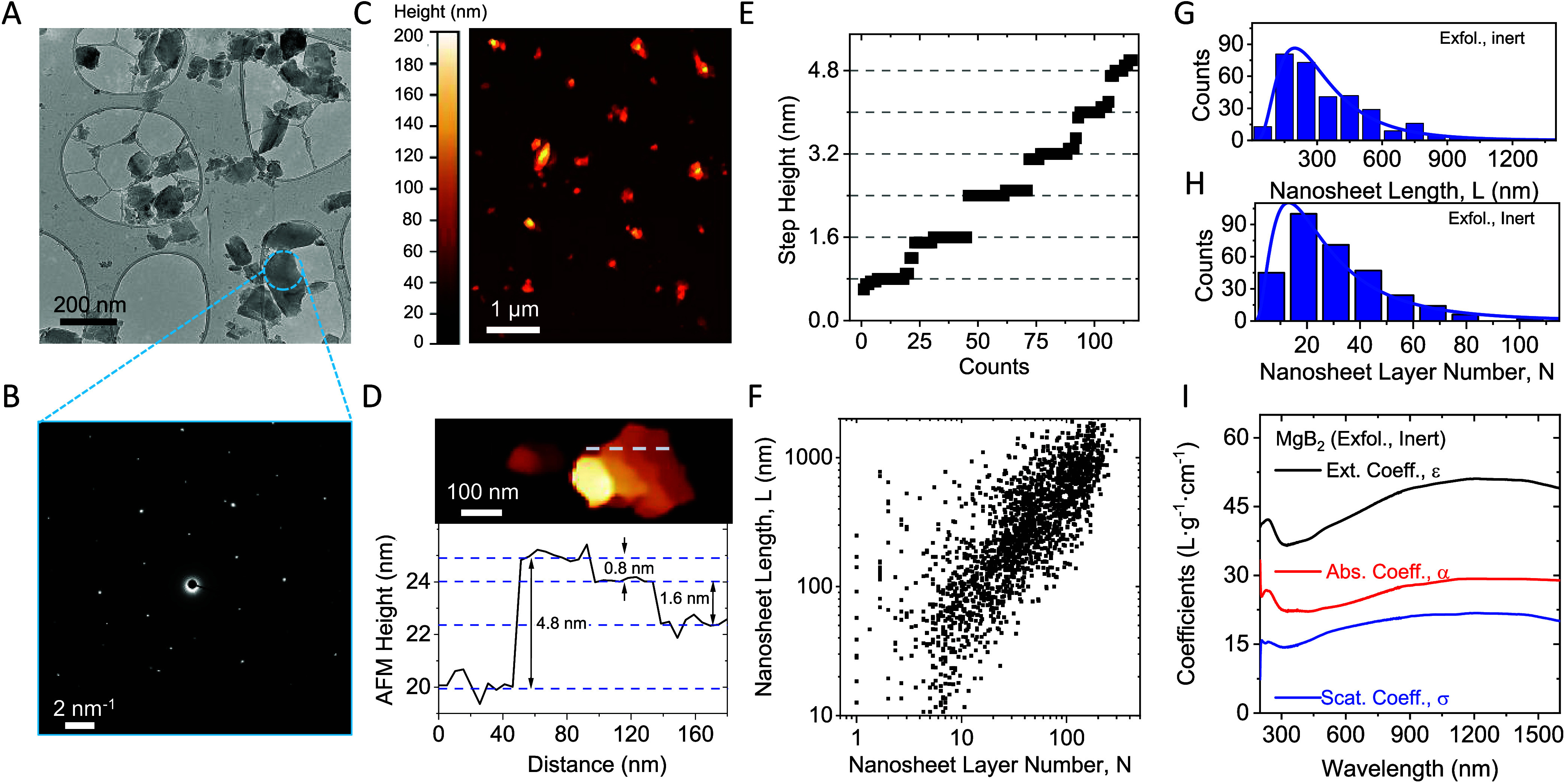
Quantitative characterization of MgB_2_ nanoplatelets prepared under inert conditions. (A) Bright-field TEM image of drop cast MgB_2_ nanoplatelets. A distribution of different sizes is observed, similar to the nanomaterials observed in the SEM. (B) SAED for the triangular particle in panel (A). The probed region is indicated by the dashed circle and demonstrates the reasonable crystallinity of the nanomaterial. (C) Overview AFM image of MgB_2_ nanosheets. Particles of a similar size and shape to those seen in TEM are observed. (D, E) Conversion of the apparent AFM height into the LPE MgB_2_ layer number using line profiles on nanoparticles with suitable steps and terraces. An example is shown in panel (D), while the statistical analysis of suitable steps is presented in panel (E). The data are sorted in ascending order, revealing discrete steps of ∼0.8 nm. (F) Scatter plot of the overall nanosheet size and thickness distribution measured using statistical AFM. The data includes the size and thickness of over 1500 nanoparticles with an average length/thickness aspect ratio of ∼12.9. (G, H) Histograms of the lateral nanosheet size (G) and the layer number (H) from statistical analysis of line profiles on individual nanosheets. (I) Optical extinction (black), absorbance (red), and scattering (blue) coefficient spectra for MgB_2_ nanoparticles exfoliated under inert conditions.

Due to the strong impact of ambient oxygen on the nanomaterial composition, only inert processed nanosheets were subjected to further analysis. In order to assess the preservation of crystallinity in the exfoliated material, samples were deposited via drop casting under inert conditions and subsequently analyzed using TEM, as well as selected-area electron diffraction (SAED) measurements. Bright-field TEM imaging shows different sizes of MgB_2_ particles (Figure 3A). SAED measurements performed on diverse particles reveal that thicker particles typically exhibit crystalline properties, whereas thinner particles tend to possess amorphous characteristics (Figure 3B; see SI, Figure S1 for additional examples).

To gain further insights into the dimensions of the produced MgB_2_ nanosheets, AFM was employed to quantitatively analyze the nanosheet size and thickness distributions. For this purpose, diluted ink samples were deposited on Si/SiO_2_ substrates, as described in more detail in the SI, and subjected to statistical analysis. An overview AFM image is shown in Figure 3C, presenting nanosheets of similar size and morphology as those observed by electron microscopy. As the nanosheet size and thickness distribution of a standard sample are typically very broad, images of the same region have been measured at different resolutions to account for pixelation effects. Additionally, reported corrections based on the correlation of comprehensive TEM and AFM statistics on samples analyzed using the same type of AFM cantilevers as in this work have been applied to account for tip broadening.

In order to convert the measured apparent thickness into nanosheet layer number, factors such as solvent residues, measurement parameters, and distinct interactions between the tip and the nanosheet or substrate surface need to be taken into account.^44−47^ Thus, the measured height (*t*) is proportional but not identical to a multiple of the crystallographic interlayer distance of the nanosheets. To this end, the measured height from AFM can be converted into the nanosheet layer number (*N*) by careful analysis of step heights of incompletely exfoliated nanosheets.^48−50^ An example of a nanosheet with steps of different layer numbers is shown in Figure 3D. The measured AFM profile across the nanosheet, as indicated by the dashed line, shows steps with a relative height of a multiple of the apparent AFM monolayer thickness.^51^ Statistical analysis on the step height extracted from many nanosheets reveals the measured step height to cluster in multiples of 0.8 nm (Figure 3E), a value that can be taken as the apparent thickness of the thinnest possible MgB_2_ structure, which we term a monolayer, and assume to consist of a combination of individual boron and magnesium layers. Thus, this value can be used to convert the measured height into the number of monolayers per nanosheet (*N*).

The above-mentioned considerations, in combination with careful statistical analysis of large data sets, enable us to reliably determine the nanosheet size and thickness distribution. This is a powerful method for further elaboration of theoretic concepts and subsequent correlation of such to measured nanosheet properties either in dispersion^9,52^ or in deposited films.^53^ The result of the statistical evaluation of the nanosheet length and thickness for more than 1000 individual MgB_2_ nanosheets is presented in Figure 3F. This scatter plot of the length, *L*, versus layer number, *N*, of individual nanosheets shows that thin nanosheets tend to be small and vice versa, which is a typical observation for nanomaterials prepared by sonication-assisted liquid-phase exfoliation.^32^ Histograms for the nanosheet length (Figure 3G) and layer number (Figure 3H) show the typical log-normal distributions for the nanosheet dimensions. The AFM data for the nanosheet length can be compared to the size information from SEM measurements (Figure 1I). The individual datasets agree reasonably well: an average nanosheet length of <*L*>_AFM_ = 349 ± 11 nm is found from statistical AFM measurements, and <*L*>_SEM_ = 387 ± 10 nm has been extracted from SEM. The average nanosheet layer number <*N*> could only be determined from AFM measurements and is found to be 30 ± 1 layers from statistical analysis. In addition, taking the monolayer thickness as the *c*-axis lattice parameter (0.35 nm) allows one to calculate the mean nanosheet aspect ratio (length/thickness) to be 12.9. Further, we find a power law relation between the mean nanosheet area and the average layer number consistent with previous reports^9,32,54^ (see SI, Figure S2).

In further analysis, the yield of the exfoliated material was determined to be 21% of the initial mass by gravimetric weighing of a known volume of the filtered nanomaterial. While the yield of the exfoliated material appears low, it should be noted that the fraction removed as unexfoliated material after the first centrifugation step can be used for subsequent exfoliation steps, as demonstrated previously for other material systems.^14,55,56^ Knowledge of the nanomaterial yield holds significance for many reasons, as it provides quantitative insights into the efficiency of the exfoliation process, aiding process optimization, and hence cost-effectiveness. Therefore, coefficient spectra of exfoliated nanomaterial have been determined for optical extinction, ε(λ), and absorbance, α(λ), using nanomaterial inks at known concentrations. Additionally, this allows extraction of the wavelength-dependent scattering coefficient σ(λ) from the relation σ(λ) = ε(λ) – α(λ).^57^ The coefficient spectra are shown in Figure 3I, with each showing broad featureless behavior consistent with the metallic nature of MgB_2_. Knowledge of the optical coefficients is valuable as the spectra not only enable fine-tuning of the MgB_2_ ink concentration for unknown samples, facilitating swift and reliable standard measurements, but also provide qualitative insights into the nanomaterial size and thickness distribution from the scattering contribution to the extinction spectra.

### Deposition of MgB_2_ Thin Films

While the capability of preparing nanomaterials without altering their structure or chemical composition is important, depositing them into thin films with controllable morphology while preserving their structural integrity holds similar significance. To this end, three different deposition techniques were tested: vacuum filtration, spray coating, and a modified Langmuir–Schaefer (LS) deposition, with the expectation that each method yields slightly different film morphology. This allows us to study the influence of morphology on the electrical properties of the films, as well as their impact on the materials’ spectroscopic response (i.e., indications for material decomposition). For this purpose, the same spectroscopic methods were applied to the deposited nanomaterial as used for the initial nanosheet characterization mentioned above. A combination of optical and electron microscopy, EDX, and IR spectroscopy as well as *I*–*V* measurements are shown and discussed for each deposition technique in the SI (Figure S3). Experimental details are given in the Methods/Experimental Section (see SI). Based on the results from both microscopy and spectroscopy, Langmuir–Schaefer deposited films appear to be more uniform and demonstrate fewer signs of material decomposition when compared to films fabricated by filtration and spray coating (Figure S3).

**Figure 4:**
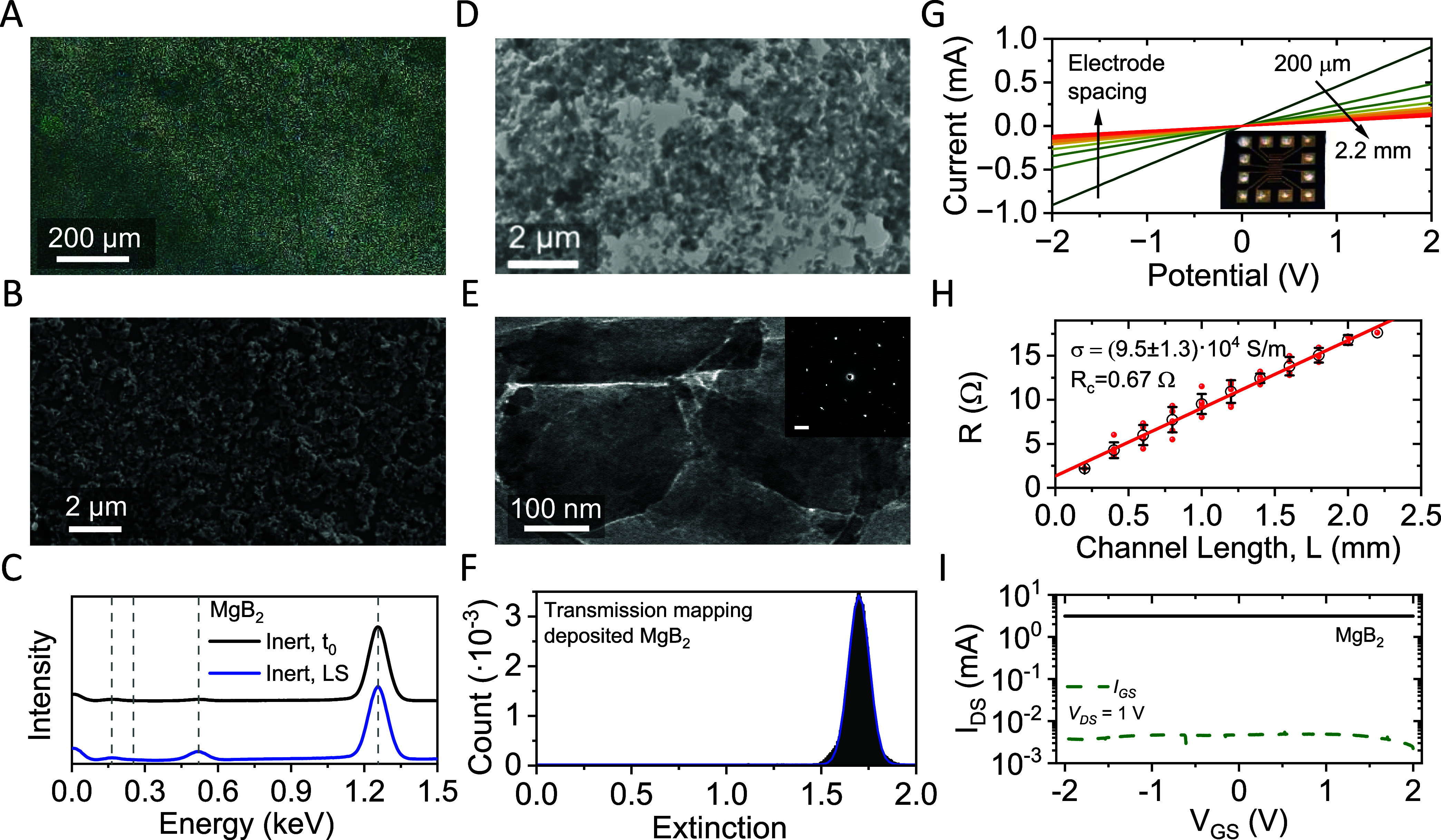
Characterization of MgB_2_ nanoplatelet thin films made by Langmuir-type deposition. (A, B) Optical micrograph (A) and SEM image (B) of a MgB_2_ thin film after a single deposition. The sample is homogeneously covered with MgB_2_ nanoplatelets. (C) EDX spectra of a deposited thin film (blue) in comparison with the freshly inert exfoliated nanomaterial (black). Note that minimal oxidation is observed after the deposition. (D, E) TEM characterization of Langmuir-type deposited inert MgB_2_ on a TEM grid. Nanoparticles are deposited over the entire grid, with sparser regions around the holes in the carbon film (D). Nanosheets show edge-to-edge alignment in the film with a partial overlap (E). SAED measurements on an individual nanosheet reveal high crystallinity after the deposition (E, inset). (F) Extinction histogram from white light transmission scans mapped over a 1 cm × 1 cm sample area deposited on quartz glass. The peak is well described by a single Gaussian distribution (blue line). Note that the single modality is indicative of a homogeneous thickness profile across the sample area. (G, H) *I*–*V* characteristics (G) and network resistance (H) as a function of channel length, L, for a MgB_2_ thin film (average thickness of 570 nm). The sample was characterized using patterned gold top contacts, as shown in the photograph (G, inset). To confirm the homogeneity of the deposited nanomaterial film, all possible electrode combinations have been tested, resulting in an average thin film conductivity of (9.5 ± 1.3) × 10^4^ S/m (F) and a contact resistance of 0.67 Ω. (I) Transfer characteristics of an electrolytically gated MgB_2_ thin-film transistor after encapsulation with the gate leakage shown as the dotted line. No charge modulation is observed, demonstrating the metallic characteristics of the nanomaterial.

Consequently, the following discussion focuses on the attributes of nanosheet networks prepared by Langmuir-type deposition. It is worth noting that the modified Langmuir–Schaefer deposition technique employed in this study is currently experimental and will undergo further refinement in the future. In our methodology, the nanomaterial is introduced into a liquid–liquid interface (water–hexane), leading to a reduction in interfacial energy at the injection point. This initiates the self-assembly of a tiled nanomaterial layer at the interface once a sufficient amount of the material is introduced. Images captured through optical microscopy and SEM of a transferred self-organized MgB_2_ nanosheet layer are presented in Figure 4A,B.

In further analysis of the nanosheet networks, EDX spectra were acquired on LS films of the deposited nanosheets. The data are shown in comparison to measurements on freshly exfoliated nanosheets drop cast in an argon atmosphere (Figure 4C). According to the EDX measurements, only slight oxidation of the material occurred during the LS deposition process, suggesting a ratio of Mg/B/O of 1:2:0.8. While care should be taken about the suggested stoichiometry determined by EDX spectroscopy, qualitative trends can be used to compare the oxygen content in fabricated samples. To this end, EDX and IR spectra have been acquired on samples after filtration, spray coating, and Langmuir-type deposition. Spectra acquired on filtered and sprayed nanosheets are shown in the SI (Figure S3). The measurements suggest that less oxygen was introduced in the LS-deposited films compared to filtered (EDX Mg/B/O ratio of 1:1.5:2.7) or spray deposited (1:2.8:1.1) nanosheets. For additional results and a detailed discussion, see SI, section 3.

In order to study crystallinity and any potential preferred orientation effects in the deposited nanomaterial, Langmuir-type deposition was performed on a TEM grid and subjected to SAED measurements (Figure 4D,E). The sample displays homogeneous coverage with sparser regions close to holes in the grid. Nanosheets exhibit edge-to-edge orientation with some edges partially overlapping (Figure 4E). SAED measurements on an individual nanosheet demonstrate that the crystallinity is not affected during the deposition steps (Figure 4E, inset). However, no indication of any preferential orientation of the nanosheets within the network is discernible (see SI, Figure S1G,H for additional data).

While microscopy indicates a uniform distribution of the nanomaterial on both the micro- and mesoscales, it would be useful to quantify the homogeneity of the nanomaterial coverage across the entire substrate. This can be assessed through spatially resolved transmission scanning. To achieve this, a MgB_2_ network deposited onto a glass substrate was subjected to transmission scanning (pixel size ∼5 μm). The scanner response was converted into extinction, as elaborated in more detail in the Methods/Experimental Section. The results are presented in the form of an extinction histogram (Figure 4F), displaying a Gaussian bell shape. We assess the sample homogeneity via the coefficient of variation (ratio of the standard deviation to the mean). For the distribution histogram in Figure 4F, a coefficient of variation of 0.05 is found, which signifies that the standard deviation of the thickness distribution is 5% of the mean thickness. The thickness and morphology of the deposited nanosheet network is hence considered as homogeneous across the entire substrate when probed at a length scale of ∼5 μm.

To test the electrical conductivity of the LS-type deposited nanosheets, gold electrodes were deposited on top of an MgB_2_ nanosheet network in transmission line geometry. Contact pads were reinforced with silver paint to provide better contact with the needle probes (see the inset in Figure 4G). The average *I*–*V* curves measured for an array of different electrode spacings are shown in Figure 4G. Ohmic behavior is observed for all measurements on the Langmuir-type film, indicating that the nanosheets retain their semimetallic character after exfoliation and deposition. Crucially, this is not the case for networks deposited by filtration (see SI, Figure S3M), spray coating (see SI, Figure S3N), or for LS-type samples after exposure to ambient conditions (see SI, Figure S4). The network resistance was measured for each electrode separation and is plotted versus channel length, *L*, in Figure 4H. The distribution of resistance values is indicated by red dots in Figure 4H, corresponding to individual *I*–*V* measurements. The mean resistance for each channel length is given by black circles, with error bars representing the standard deviation. A linear relationship is found consistent with *R* = 2*R*_C_ + *L*/(σ*Wt*), where *R*_C_ is the contact resistance, *W* = 2.4 mm is the channel width, and *t* = 570 nm is the film thickness. Fitting shows the film conductivity to be σ = (9.5 ± 1.3) × 10^4^ S/m and gives a contact resistance of *R*_C_ = 0.7 Ω (*R*_C_*W* = 1.7 Ω m). This conductivity is quite high for solution-processed nanosheet networks and is competitive with the state-of-the-art for printed graphene films (∼10^5^ S/m).^19−23^ However, it is somewhat below the reported conductivities for MXene films (∼10^6^ S/m)^58^ owing to the far lower aspect ratio of the diboride nanosheets,^19^ and lower than networks of metallic nanoplatelets (∼10^7^ S/m)^59^ which, following sintering and filament formation, are not separated by a van der Waals gap.

Since linear *I*–*V* curves can also be indicative of bulk-limited semiconducting channels, we performed transistor measurements to further confirm the semimetallic behavior of the MgB_2_ network. An electrolytically gated thin-film transistor was fabricated from a four-times-deposited MgB_2_ nanosheet network in a displaced-gate electrode geometry, where an ionic liquid connects the channel to the gate (see SI, Methods section, for further details). A gate bias causes the ions to separate and form a double layer on the gate electrode and also throughout the internal surface area of the nanosheet network. For a semiconducting network, this causes an increase in the current density as carriers fill the network to balance the ionic charge. However, there is no current modulation observed in the transfer characteristic in Figure 4I, which is further confirmation of the semimetallic electrical properties of these networks.

### Stability of the Nanomaterial

As preliminary measurements strongly suggest that the nanomaterial is prone to degradation in ambient conditions, the stability of the nanosheets exfoliated in inert conditions was studied both in the dispersion and after Langmuir-type deposition to form a film. In the Supporting Information, we report on preliminary experiments to demonstrate the buildup of Langmuir-type multilayers using multiple iterations of the deposition process to increase the film thickness (see SI, Figure S5). For the purpose of optical measurements on metal diboride thin films, four iterations of the Langmuir–Schaefer deposition have been used on optical glass to increase film thickness for a better signal-to-noise ratio.

**Figure 5:**
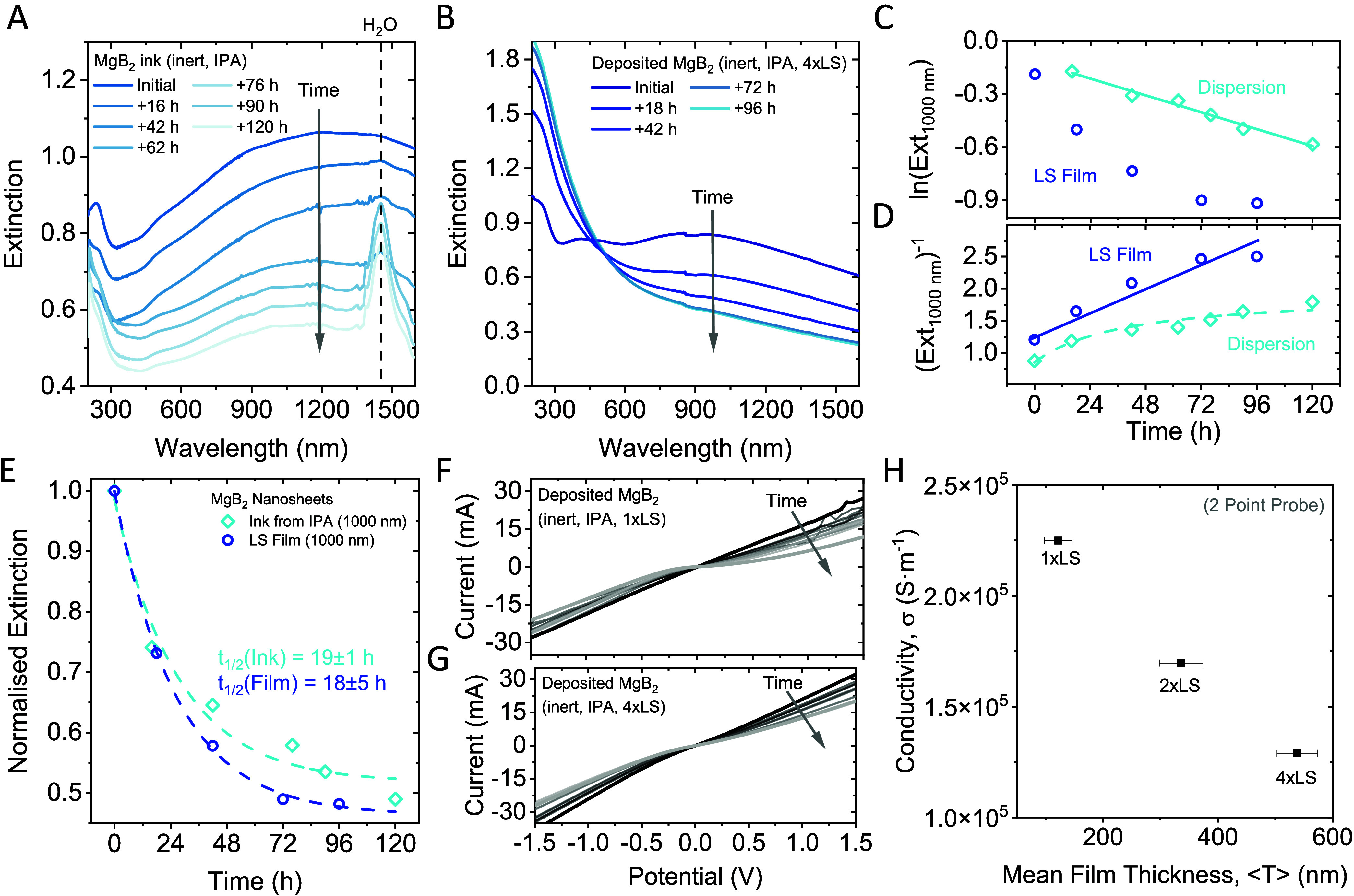
Nanomaterial stability. (A, B) Change in the extinction of liquid exfoliated MgB_2_ nanoplatelets in dispersion (A) and after thin film deposition on optical glass (B) as a function of time exposed to ambient conditions. In both cases, a systematic decrease in the overall response is observed for all wavelengths above 450 nm, while the signal below 450 nm undergoes changes in the spectral profile and shows an increased optical density in the UV region, which is indicative of oxide formation. Further, a peak that can be attributed to water forms over time in the nanosheet ink. (C, D) Kinetic plots for rate law analysis of the extinction measurements shown in panels (A, B). Panel (C) shows the change in the natural logarithm of the optical density at 1000 nm, and panel (D) shows the inverse optical density at 1000 nm as a function of time. While the lines indicate a reasonable agreement with a first-order rate law for the dispersed nanomaterial (C) and a second-order rate law for the deposited material (D), the analysis is not unambiguous and is discussed in further detail in the SI. (E) Change in the extinction of the nanosheet ink and thin film at 1000 nm as a function of time. The data can be described by an empirical exponential fit, which allows determination of the material’s half-life. The data for both individual data sets agrees well, and fitting suggests a nanomaterial half-life of 19 ± 1 h and 18 ± 5 h for the nanosheet ink and thin film, respectively. (F, G) *I*–*V* characteristics as a function of time for two sets of samples measured after deposition of a single layer (F) and after deposition of 4 layers (G). In both cases, similar systematic changes are observed: the initial ohmic response changes to a more rectifying transport behavior, which is consistent with the formation of an oxide layer. (H) Conductivity of the same MgB_2_ films measured as a function of the mean film thickness determined by profilometry at different points across the substrate. A decrease in the conductivity is observed with increasing film thickness, which is counterintuitive, and may be attributed to surface oxidation of the nanomaterial during the processing steps.

Samples were exposed to ambient conditions after preparation, and extinction spectra were acquired as a function of exposure time, ranging from 0 to ∼100 h. For the deposited thin films, additional changes in the *I*–*V* characteristics were recorded over time. The results from the photospectroscopic measurements are shown in Figure 5A,B for the nanosheet dispersion and a thin film, respectively. In both cases, systematic changes are observed as a function of the exposure time to ambient conditions. The dispersion (Figure 5A) shows a steady change over the entire accessible spectroscopic response. In addition, the initial peak at ∼220 nm undergoes a systematic change, in line with an electronic contribution that can be assigned to oxidized magnesium species, and a progressive evolution of a water peak at ∼1450 nm. The latter demonstrates the initial absence of water in the solvent and how the solvent acquires ambient water over time. The change in the peak at 220 nm as well as the decrease of the overall optical density can be attributed to oxidation of the nanomaterial. We can rule out effects from aggregation and sedimentation of the nanomaterial, as the dispersions were refreshed by sonication prior to each measurement, and no increase of the scattering contribution is observed in the extinction spectra over time.

The Langmuir–Schaefer deposited nanosheet film (Figure 5B) shows similar time-dependent changes in the photospectroscopic profile to those of the dispersion. However, the changes to the peak in the UV region are more pronounced in the film compared to the ink. We presume that this is because of direct exposure of the film to ambient conditions while the nanosheets in dispersion are embedded in a solvent matrix, slowing down the degradation to the diffusion limit.

However, such behavior should be evident from kinetic analysis of the spectra. For this purpose, time-dependent changes to the spectra were plotted according to a first- (Figure 5C) and a second-order (Figure 5D) rate law. Here, the change in the initial MgB_2_ concentration is represented by the extinction at 1000 nm, which is solely attributed to the electronic response of the nanomaterial.

When plotted as a natural logarithm of the optical density versus time, in line with a first-order rate law (Figure 5C), the data acquired for the dispersion follows a linear trend, as expected for a diffusion limited decomposition reaction. This is in contrast to the spectra of the deposited film, where no linearization is observed. In contrast, when plotted as inverse optical density over time (Figure 5D), the thin-film data are linear, supporting a second-order rate law for the decomposition of the deposited nanomaterial. However, while additional data was collected on other metal diborides (CrB_2_ and ZrB_2_), the observed trends do not allow for an unambiguous rate analysis of the decomposition chemistry. Further details are given in section 6 of the SI. For further analysis, we present an empirical fit to a single exponential function

, which enables one to estimate the portion of reacted (PoR) material from the amplitude as PoR = ε_1_/(ε_0_ + ε_1_) (Figure 5E). Approximating the data from the UV-vis measurements on an LS-deposited MgB_2_ nanosheet film to infinite time scales suggests that 54 ± 1% of the nanomaterial decomposes with an estimated macroscopic nanomaterial half-life of 18 ± 5 h for storage in ambient conditions.

The data from spectrophotometric measurements already gives reasonable insights into the kinetics of the nanomaterial decomposition, as previously demonstrated on other 2D nanomaterial systems.^9,49,60,61^ However, it is expected that the decomposition also has an impact on the physical properties of the nanosheets, such as their conductivity. It was not possible to correlate conductivity measurements with optical measurements taken on the nanosheet dispersion despite the attempt to deposit nanomaterial from similarly aged dispersions, as the amount of material was insufficient. However, it was possible to measure changes in the conductivity on deposited nanosheet networks over time.

While ohmic transport is observed for multiple depositions (see SI, Figure S6), we note that care must be taken when moving a previously deposited nanosheet network through the water surface, as material may be washed off due to the high surface tension (see the Methods/Experimental Section for additional details).

To further study the nanomaterial decomposition upon exposure to ambient conditions, the I–V characteristics of Langmuir–Schaefer films after a single and after four iterative depositions were acquired over time (Figure 5F,G). In both cases, the curves show a systematic change from initially Ohmic to non-Ohmic transport characteristics, in line with progressing material oxidation. Note that the electric measurements were taken on the same films as those used for studying the photospectroscopic response over time, as discussed above. This allows us to correlate the conductivity change with the change of the optical characteristics over time.

Increasing the film thickness through the adapted Langmuir-type deposition did not lead to an increase in the film conductivity in the case of MgB_2_ nanosheets (Figure 5H). We attribute this phenomenon to the air sensitivity of the nanomaterial and the increased exposure to ambient oxygen with each repetition of the deposition process for augmenting the film thickness.

**Figure 6:**
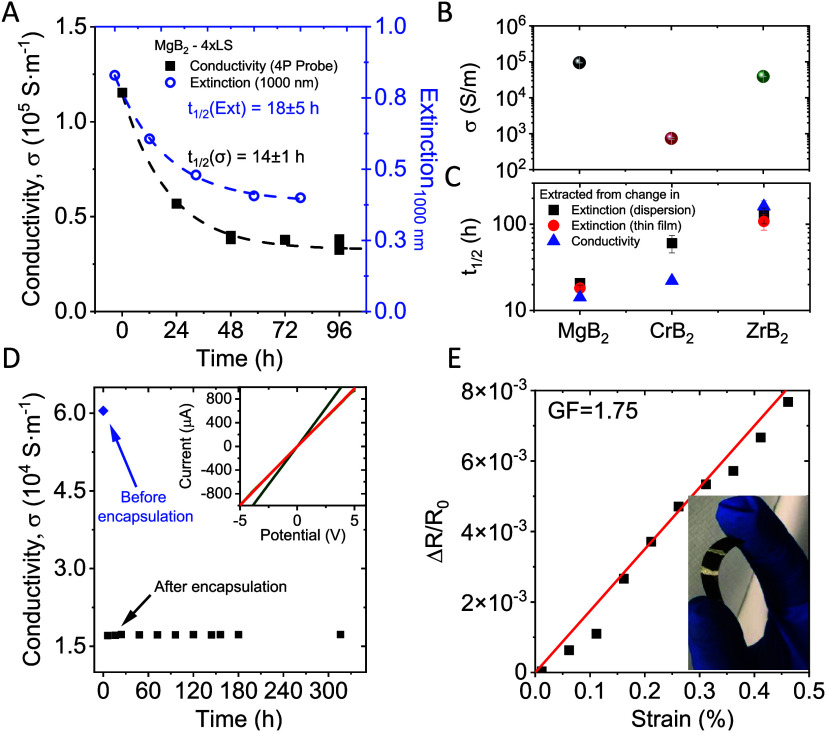
Material comparison, network encapsulation, and electromechanical characterization. (A) Changes in both the electrical conductivity and optical density at 1000 nm of MgB_2_ films (from data shown in Figure 5F,G) as a function of storage time in ambient conditions. Both data sets show a similar exponential decay, which implies the two different methods yield a comparable result for the nanomaterial decomposition kinetics. (B, C) Initial conductivity (B) and nanosheet half-life (C) for liquid deposited networks of MgB_2_, CrB_2_, and ZrB_2_ nanosheets. (D) Evolution of the MgB_2_ network conductivity before and after encapsulation. After an initial drop postencapsulation, which we attribute to changes in the thin film morphology, the conductivity stabilizes for times > 300 h. Inset: *I*–*V* curves for a MgB_2_ nanosheet thin film after 4 iterative Langmuir-type depositions. The green curve was recorded immediately postdeposition. A number of additional curves were recorded at defined time intervals after encapsulation of the film using a spray-on polymer, with the red curve corresponding to the first *I*–*V* measurement after encapsulation. (E) Fractional resistance change of a MgB_2_ film as a function of applied strain. Inset: optical photograph of MgB_2_ nanosheets deposited on PET after 4 iterations of the Langmuir-type deposition method.

In further analysis, we plot the evolution of the conductivity (black squares) and optical extinction at 1000 nm (blue circles) of a MgB_2_ nanosheet network formed by 4 successive Langmuir-type depositions over time (Figure 6A). Both data sets can be fit to the same exponential decay, as described above. However, the data acquired from conductivity measurements imply an even more severe material oxidation (PoR = 71 ± 1%) with a half-life of 14.2 ± 0.9 h.

In addition to studying the decomposition of MgB_2_ nanosheets, similar experiments have been reproduced for CrB_2_ and ZrB_2_. It is evident from the data that the other metal diborides show a comparable behavior upon exposure to ambient environment (for further details, see SI, Figures S7–S9). The conductivity of all three materials exfoliated under inert conditions and deposited as films using four iterations of the Langmuir-type deposition is shown in Figure 6B. While the conductivity of ZrB_2_ is comparable to MgB_2_, the measurements taken on CrB_2_ Langmuir-type films indicate a 100× lower conductivity.

To facilitate a comparison with MgB_2_, extinction and *I*–*V* measurements were performed on deposited CrB_2_ and ZrB_2_ networks as a function of their exposure time to ambient environment (see SI, Figures S10 and S11 for further details). All samples show systematic changes in their spectroscopic response. In all cases, the change follows an exponential decay, which can be fit reasonably well to the same equation as used for MgB_2_. This is useful, as it allows the nanomaterial’s half-life to be extracted (Figure 6C), as well as the portion of reacted material from the amplitude of the fitted exponential. While all materials show significant oxidation over time, the data indicates that a passivation layer forms based on the portion of reacted material, which is estimated to be between 45 and 65% for the three materials studied here. Further, the initially ohmic conductivity shows a transition to a more rectifying behavior as oxidation of the nanomaterial proceeds and the electrode/material contact evolves (SI, Figures S4 and S11). The results for all three materials characterized in this work are summarized in Table 1.

**Table 1:** Portion of Reacted Material (PoR) and Half-Life (*t*_1/2_) of the Nanosheets for Both Dispersed and Deposited MgB_2_, CrB_2_, and ZrB_2_ Samples; for the Thin Film Samples, the PoR and Half-Life Are Extracted from Both Extinction and Network Conductivity Data

	PoR	PoR (thin film)		*t*_1/2_	*t*_1/2_ (thin film)	
material	extinction	extinction	conductivity	extinction	extinction	conductivity
MgB_2_	46.9 ± 3.3%	54.4 ± 1.6%	71.0 ± 0.9%	20.8 ± 1.1 h	18 ± 5.0 h	14.2 ± 0.9 h
CrB_2_	57.9 ± 4.7%	n.a.	87.4 ± 1.5%	60.4 ± 13.9 h	n.a.	22.1 ± 2.0 h
ZrB_2_	64.0 ± 6.3%	77.4 ± 2.2%	92.9 ± 8.2%	127.2 ± 26.6 h	108.0 ± 22.7	160.8 ± 25 h

Due to the sensitivity of metal diboride nanosheets to exposure to ambient conditions, it is important to develop strategies to protect the nanomaterial from oxidation. While different approaches have been reported in the past,^62−64^ we demonstrate the use of a spray-on polymer as an efficient protection layer. The *I*–*V* characteristics of a MgB_2_ nanosheet thin film were tested before and after application of the polymer layer and subsequently measured as a function of time in ambient conditions for 2 weeks. In the inset of Figure 6D, we show the *I*–*V* curves of the film right before and after polymer encapsulation. After the deposition of the polymer layer, a drop in the network conductivity is observed, which we attribute to changes in the film morphology during the deposition process. Note that after this initial drop, no further change to the network conductivity is observed over a time scale of >300 h (Figure 6D).

Within the field of printed flexible electronics, conductive nanomaterials are most commonly used as an electrode material.^65^ While a high electrical conductivity is a prerequisite, it is also crucial that the electrode conductivity is not significantly affected when the device is strained. We tested the suitability of our semimetallic MgB_2_ nanosheets for this purpose by performing piezoresistive measurements on an encapsulated network fabricated by four successive Langmuir-type depositions on a PET substrate (Figure 6E). Electrical measurements as a function of strain (ε) show only slight changes to the network resistance within the studied range. The extracted gauge factor (Δ*R*/*R*_0_ = *G*ε)^66^ of *G* = 1.75 suggests that the network (or electrode) conductivity remains relatively unperturbed by applied strains. This serves to highlight the applicability of encapsulated MgB_2_ films for use as electrode materials in flexible electronics.

## Conclusions

In summary, we have demonstrated the exfoliation of three different metal diborides: MgB_2_, CrB_2_, and ZnB_2_. The sensitivity of these nanomaterials to ambient oxygen has been highlighted using the example of MgB_2_, where significant oxidation is observed upon exposure to ambient conditions, as demonstrated by UV–vis measurements, IR transmission, and EDX spectroscopy. To address this, a viable and readily accessible method for exfoliating and processing such materials into thin films under inert conditions has been demonstrated. The produced nanomaterials have been extensively characterized using a combination of advanced microscopy techniques, including SEM, TEM, AFM, and statistical analyses, to study the nanosheet size and thickness distributions have been performed.

The exfoliated metal diboride nanoplatelets have been deposited into thin films using vacuum filtration, spray coating, and a Langmuir-type deposition method, yielding different network morphologies. Promising samples from the Langmuir-type deposition were studied further, including their composition and crystallinity, by using IR transmission, UV–vis, SAED, and EDX spectroscopy, while their electrical characteristics were probed by using *I*–*V* measurements. Indeed, the Langmuir-type deposited MgB_2_ thin films in this work exhibit a high conductivity of (9.5 ± 1.3) × 10^4^ S/m and a contact resistance of 0.67 Ω if the exposure to oxygen is minimized.

As these materials are sensitive to oxygen exposure, we have studied the material decomposition kinetics and quantified the portion of reacted material and macroscopic half-life for nanoplatelets in both the nanomaterial ink and deposited films upon exposure to ambient conditions. This was achieved by tracking changes in the photospectroscopic response and *I*–*V* characteristics as a function of the exposure time. The results from both yield comparable results and suggest that more than 50% of the nanomaterial decomposes with a half-life of less than 1 day.

Finally, we demonstrated the metallic character of the deposited metal diboride nanosheet networks using electrical and piezoresistive measurements. MgB_2_ films were successfully encapsulated using a spray-on polymer to mitigate oxidation-driven degradation and electromechanically tested. The measured gauge factor of ∼1.75 for Langmuir-type deposited MgB_2_ indicates that the network conductivity is largely invariant within the studied strain range. This highlights the suitability of metal diboride films as electrode materials for flexible electronics.

## Methods/Experimental Section

### Exfoliation

In brief, 30 mg of powdered magnesium diboride (MgB2) was immersed in 30 mL of dry isopropyl alcohol (IPA) under a nitrogen atmosphere to prevent oxidation. The mixture was sonicated for 7 h in an ultrasonic bath while maintaining the temperature below 10 °C to prevent overheating. Nitrogen was bubbled through the mixture to maintain an inert atmosphere. The resulting dispersions were then size-selected through liquid cascade centrifugation (LCC) in multiple steps using gradually increasing centrifugation speeds. Unexfoliated bulk material was first separated by centrifugation at 200*g*, and the nanomaterial was further concentrated by sedimentation at 10 000*g*. The supernatant, containing oxidized material and soluble impurities, was discarded, while the sediment was redispersed in fresh solvent to obtain the nanomaterial. All steps were performed under inert conditions to maintain sample integrity, and the prepared dispersions were referred to as “stock dispersions”.

Langmuir–Schaefer-type Deposition: A custom-built setup was used for the deposition of the exfoliated nanosheets. The process began by filling a 250 mL beaker with deoxygenated water to cover the substrate completely. About 2 mL of distilled *n*-hexane was added to create a liquid/liquid interface. Nanosheet ink was then injected at this interface until a homogeneous film was formed. The substrate was lifted through the interface to transfer the nanosheet layer, dried under a nitrogen stream, and annealed at 120 °C for 2 h in an argon atmosphere to remove any residual water. For subsequent depositions on an existing layer, the substrate was briefly dipped in acetone before the next deposition step.

A detailed description of all methods, including materials characterization, is given in the Supporting Information.

## Supporting Information Available

Experimental methods, additional information on nanomaterial characterization, network performance for different deposition techniques, layer-by-layer deposition, exfoliation and analysis of CrB_2_ and ZrB_2_, degradation kinetics data, and literature comparison (PDF)
