# Supplementary material for: Inert Liquid Exfoliation and Langmuir-Type Thin Film Deposition of Semimetallic Metal Diborides
Source: ACS Nano. 2024 Oct 9;18(42):28596–608. doi: 10.1021/acsnano.4c04626 (PMC11503910; doi:10.1021/acsnano.4c04626)
Supplement: Supplementary file 1 — nn4c04626_si_001.pdf [file nn4c04626_si_001.pdf]

Supporting Information for

Inert liquid exfoliation and Langmuir-type thin film deposition of semimetallic metal diborides.

Kevin Synnatschke,[1*] Alina Müller,[1,2] Cian Gabbett,[1] Michael Johannes Mohn,[3] Adam G. Kelly,[1] Kseniia Mosina,[4] Bing Wu,[4] Eoin Caffrey,[1] Oran Cassidy,[1] Claudia Backes,[2,5] Zdenek Sofer,[4] Ute Kaiser,[3] Jonathan N Coleman[1,2*]

[1] *School of Physics, CRANN & AMBER Research Centres, Trinity College Dublin, Dublin 2, Ireland*

[2] *Chair of Applied Physical Chemistry, Heidelberg University, Im Neuenheimer Feld 253, 69120 Heidelberg, Germany*

[3] *Central Facility of Electron Microscopy, Electron Microscopy Group of Materials Science, Ulm University, Albert-Einstein-Allee 11, 89081 Ulm, Germany*

[4] *Department of Inorganic Chemistry, Faculty of Chemical Technology, University of Chemistry and Technology Prague, Technická 5, Prague 6, 166 28 Czech Republic*

[5] *Institute of Physical Chemistry, University of Kassel, Heinrich-Plett-Straße 40, 34132 Kassel, Germany*

*synnatsk@tcd.ie

*colemaj@tcd.ie

Contents

1 Experimental Methods 3

1.1 Solvent Preparation 3

1.2 Synthesis 3

1.3 Nanomaterial Preparation 3

1.4 Thin Film Deposition 4

1.5 Material Characterisation 5

2 Nanomaterial Characterisation 8

2.1 TEM Measurements 8

2.2 AFM Measurements 10

3 Network Characteristics for Different Deposition Methods 12

4 Layer by Layer Deposition of Tiled Nanosheet Networks 19

5 Exfoliation and Langmuir-Schaefer Deposition of CrB_2 and ZrB_2 21

6 Decomposition Kinetics for MgB_2, CrB_2 and ZrB_2 25

7 Comparison to Previous Reports 29

8 References 31

1 Experimental Methods

1.1 Solvent Preparation

Molecular sieves (10 Å) were dried at 300°C in vacuum overnight.

Isopropanol was dried over molecular sieves for 2 days and distilled prior to use (bath temperature: 120°C). The distillate was saturated with nitrogen by bubbling nitrogen gas, dried over sulfuric acid, through the solvent for 12 h.

1.2 Synthesis

Due to significant batch to batch variations of the material composition (oxygen content) for purchased powder (Sigma Aldrich, order number 553913-5G), MgB_2 powder was synthesised by solid-state reaction as described below.

Powdered MgB_2 starting material was synthesized by the direct reaction between Mg and B power in stochiometric ratio under high vacuum in a quartz ampule with an alumina liner. The pressed pellet from the homogeneous powder mixture was heated to 800°C for 50 hours. The reaction product was ground, pressed into a new pellet and heated to 800°C for additional 50 hours under high vacuum.

1.3 Nanomaterial Preparation

Liquid Phase Exfoliation (LPE). 30 mg of powdered MgB_2 starting material was immersed in 30 mL of dry IPA in a nitrogen atmosphere. For exfoliation, the mixture was sonicated for 7 h using a Branson ultrasonic bath (CPX2800-E, 130W) in a 50 ml round bottom flask. During exfoliation, nitrogen was bubbled through the liquid to maintain an inert atmosphere. The bath temperature was kept below 10°C at all times by constantly exchanging the water in the bath with ice-cooled water from a reservoir to avoid effects from sample heating. A standard aquarium pump was used for this purpose. Dispersions were size-selected by LCC in multiple steps by iteratively increasing centrifugation speeds. Exfoliated nanomaterial was isolated from unexfoliated material by previously established centrifugation techniques.[1]

To this end, the dispersions were sealed in a centrifuge tube in a nitrogen atmosphere using thermoplastic thin film (parafilm® M, order number: 52858-076, VWR). A Hettich Mikro220R centrifuge equipped with a fixed-angle rotor (1195A, 1.5 mL polypropylene tubes) was used for centrifugation. For removal of unexfoliated material, as sonicated dispersions were subjected to 2 steps of centrifugation. Initially, the samples were centrifuged at 200 *g* to separate unexfoliated bulk material from the nanomaterial. The supernatant obtained after this step was then subjected to a subsequent centrifugation at higher acceleration, while the sediment was discarded. In the second centrifugation step, the nanomaterial was concentrated by sedimentation at 10k *g*. To this end, the resulting supernatant was discarded and the sediment was re-dispersed in a reduced volume of fresh solvent. Following this protocol additionally serves to eliminate oxidised material, and soluble impurities from the nanomaterial as these typically remain in the supernatant. All decanting steps were carried out under inert conditions. Dispersions prepared according to this protocol are referred to as stock dispersion.

1.4 Thin Film Deposition

Substrate treatment. Substrates used for the AFM measurements and the film deposition methods were cleaned prior to the deposition. For this purpose, substrates were immersed, and sonicated in acetone, isopropanol, and ultrapure water for 10 minutes, respectively.

Gravimetric filtration. The concentration of the nanomaterial in dispersion was determined by filtration of a known volume of nanosheet dispersion through aluminium oxide membranes (Whatman Anodisc, 0.02 µm pore size) and subsequent washing with 200 ml Isopropanol. The membranes were dried at 60°C overnight and weighed 3 times before and after the filtration. The deposited material was allowed to dry at 60°C in inert atmosphere prior to weighing.

Spray deposition. Spray coating was performed with the Janome JR 2300N mobile gantry at room temperature. The nanomaterial dispersion with an approximate concentration of ~1 g/L, is referred to as ink in the following. The ink was sonicated for 10 minutes prior to use, to prevent any impact from material aggregation during the spray deposition. The spray coater was equipped with a Harder & Steenbeck Infinity spray gun with a 0.4 mm ventilation needle and a stand-off distance of 100 mm between the nozzle and substrate was used. The N_2 backpressure was set to 4 bar.

Langmuir Schaefer-type deposition: For the Langmuir Schaefer-type deposition, a custom-built setup, was used as published recently. A 250 ml beaker was filled with deoxygenated water until the water level was completely covering the substrate on the substrate holder. Approximately 2 ml of distilled *n*-Hexane was added to the water in the beaker to create the liquid/liquid interface. The nanosheet ink (c_{Ink} = ~1 g/L) was injected to the liquid-liquid interface using a Pasteur pipette until a homogenous film had formed and the substrate was lifted through the liquid/liquid interface to transfer the nanosheet layer onto the substrate. The wet substrate was allowed to dry at room temperature in a nitrogen stream. Dry films were annealed at 120°C for 2 hours in an argon atmosphere prior to subsequent depositions, or characterization to remove residual water from nanosheet junctions and interfaces. For the deposition of additional layers on top of an existing Langmuir-type network, we briefly dip the substrate in acetone prior to the deposition.
Following this procedure has two effects:

1) The interfacial tension when moving through the water surface is decreased, which is important to avoid material being peeled off.

2) The surface energy of the film decreases due to the removal of organic surface contaminations (*e.g.*, introduced during annealing inside the glovebox).

1.5 Material Characterisation

Atomic Force Microscopy (AFM). For AFM measurements, a Bruker Multimode 8 scanning probe microscope was used in ScanAsyst mode (non-contact) in air under 4ambient conditions using aluminium coated silicon cantilevers (OLTESPA-R3). The concentrated dispersions were diluted with isopropanol to optical densities <0.1 at 300 nm. A drop of the dilute dispersion (10 µL) was flash-evaporated on pre-heated (175°C) Si/SiO_2 wafers (0.5x0.5 cm^2) with an oxide layer of 300 nm. After deposition, the wafers were rinsed with ~15 mL of water and ~15 mL of isopropanol and dried with compressed nitrogen. Typical image sizes ranged from 15x15 μm^2 for larger nanosheets to 3x3 μm^2 for small nanosheets at scan rates of 0.2-0.5 Hz with 1024 lines per image. Previously published length corrections were used to correct lateral dimensions from cantilever broadening.[2]

Spectrophotometry. Optical extinction and absorbance measurements were carried out, using a Perkin Elmer 1050 spectrophotometer in quartz cuvettes. For absorbance measurements, cuvettes were placed in the centre of an integrating sphere. The optical density of the dispersions was adjusted to 0.4−0.8 at the peak. Measurements of both extinction and absorbance spectra on the same dispersions

allows to calculate the corresponding scattering spectra (Ext−Abs). All spectra were acquired with 1 nm increments and 1s integration time.

Optical bright field microscopy. The brightfield microscopy images were acquired with an Olympus DSX10-UZH optical microscope either through a 50x or 100x magnification objective lens.

Scanning Electron Microscopy (SEM). SEM images were acquired with a Carl ZEISS Ultra Plus SEM, using a GEMINI® objective lens with different acceleration voltages but not higher than 10 kV at 2.5×10^{-9} mbar. The images were measured with a dual (upper and lower) detector system consisting of collector-, scintillator-, light guide-, and photomultiplier units for secondary electron imaging (SEI). Energy Dispersive X-ray (EDX) analysis was performed at 15 kV, at 9 mm working distance.

Attenuated Total Reflection Infrared Spectroscopy (ATRIR). ATRIR measurements were carried out with a PerkinElmer Spektrum 100, equipped with a Diamond KRS-5. For the ATRIR measurements of pristine nanomaterial, dispersions were allowed to dry at room temperature in an argon atmosphere. Measurements were performed on the dry powder in ambient atmosphere. To measure the ATRIR of deposited films, material was scraped off the substrate and collected for the measurements. All spectra were acquired with 128 scan accumulations.

Profilometry. The thickness of deposited films was measured on a Veeco Dektak 6m. Multiple spots were measured and averaged for each film. A stylus force of 1 mg was applied.

Electrical measurements. I/V characteristics were studied in ambient conditions using a Keithley 2612A source meter connected to a probe station. For the initial characterization, conductivity measurements were performed using equally dimensioned and distanced silver electrodes. For this purpose, multiple electrodes were painted onto deposited films using a copper wire (⌀ = 0.2 mm). Electrode dimensions were determined individually, using optical microscopy. After optimization of the deposition, ultrasonically cleaned Si/SiO_2 chips (~2x2 cm) with patterned Ti/Au electrodes (2nm Ti, 60 nm Au using a Temescal FC2000 metal evaporation system) were used to facilitate electrical measurements. Samples were annealed and sealed in an argon atmosphere for transportation and unpacked immediately before the measurements for acquiring the final data.

Transmission Electron Microscopy (TEM). For structural investigation by TEM, dispersed MgB_2 nanosheets were diluted with isopropanol and drop-cast on lacey carbon TEM grids in a N_2 purged glovebox. The samples were then sealed in vacuum bags for sample shipment. Bright-field / dark-field TEM and selected-area

electron diffraction (SAED) were performed with a Thermo Fisher Talos 200X TEM at an acceleration voltage of 80 kV.

Transmission mapping. For homogeneity analysis of deposited thin films, transmission mapping was performed. To this end, a sample was deposited on a glass slide, and scanned using an Epson Perfection V850 Pro bed scanner with a resolution of 4.800 dpi. An empiric calibration curve (eq. 1) from scans on transmission filters was used to convert the scanner response to optical transmission. Here, T is the transmission of the sample and S is the signal output from the scanner.

$$T = -0.01622 + (4.2922 \cdot 10^{-6})S + (1.6598 \cdot 10^{-10})S^2 \qquad \text{(Eq. 1)}^{3}$$

Thin film transistor (TFT) fabrication. Following the deposition of the MgB_2 network, we deposited Ti/Au (5 nm/95 nm) electrodes by evaporation (FC-2000 Temescal Evaporator) through a shadow mask with L = 200 µm and W = 2.4 mm with a displaced gate geometry where the gate electrode is 10× the area of the channel. We utilize the ionic liquid 1-ethyl-3-methylimidazolium bis(trifluoromethyl-sulfonyl)imide (EMIM-TFSI, Sigma-Aldrich) to modulate the current in the channel following the procedure described in Ref.[4] A small droplet of the ionic liquid is pipetted onto the device covering both the channel and the gate. As the liquid penetrates all parts of the network, the current in all nanosheets within the channel volume can be modulated. The electrical characterisation is performed using a Keithley 2612A dual-channel source measuring unit. The transfer characteristics were taken in a window between -2 and +2 V, with a drain-source voltage of 0.5 V, and with a scan rate of 50 mV/s.

Nanomaterial encapsulation. For encapsulation of the nanomaterial thin films, we demonstrate a facile, proof-of-concept spray-on polymer using a commercially available spray-on plaster (composed of Dimethyl ether, Ethyl acetate, Pentane, Acrylates/Dimethylaminoethyl Methacrylate Copolymer). For this initial demonstration, we use three sequential depositions of ~1-2 s spray time in close-proximity (~5 cm) to the nanomaterial network. After each deposition, we dry the polymer film at 60 °C for 5 min on a hotplate in ambient conditions before the next spray cycle was performed. The encapsulated material was then annealed at 120 °C for 1 h to remove residual solvent. A control sample was fabricated on glass following the same protocol to determine the thickness of the encapsulating layer. Profilometry on different areas of the substrate indicated an average layer thickness of 8.8 µm.

Electromechanical measurements. Sensors were tested using a Zwick Z0.5 ProLine Tensile Tester (100 N Load Cell). The films were contacted using silver

wires attached with silver paint directly on the MgB_2 film. Sample dimensions were approximately 5 mm × 25 mm with PET thickness of 1 mm. Sensors were conditioned by sawtooth profile strain cycling before testing to account for unrepresentative initial electrical responses due to reorganization of the nanosheet network. The resistance was measured using a Keithley KE2601 Source meter controlled by a 2-probe LabView program. Electromechanical measurements were made using a maximum strain amplitude of 0.5% at a strain rate of $0.5\%\cdot s^{-1}$.

2 Nanomaterial Characterisation

2.1 TEM Measurements

To validate the structural integrity of the nanomaterial, exfoliated nanosheets were studied by TEM measurements. Figure S1 shows results for measurements on material collected from the sediment (Figure S1A-B), and the supernatant (Figure S1C-D) after centrifugation of the stock dispersion. The centrifugation boundaries for collecting the nanomaterial were chosen to isolate exfoliated nanosheets from impurities and oxidised material, which can be analysed by SAED measurements. While predominantly crystalline and polycrystalline nano-platelets are found in the fraction isolated in the sediment (Figure S1B), the material remaining in the supernatant is bereft of crystallinity.

Further, similar measurements were performed on nanomaterial deposited on a TEM grid using the Langmuir-type deposition technique (Figure S1E-H). Bright-field and dark-field imaging reveals nanosheets arranged in a mosaic-like network (Figure S1E-F). SEAD measurements on this network provided insights into potential structural alterations and the alignment of nanosheets in relation to their neighbours (Figure S1G-H). While reflexes from the network are evident, no preferential alignment of the nanomaterial in the network is observed.

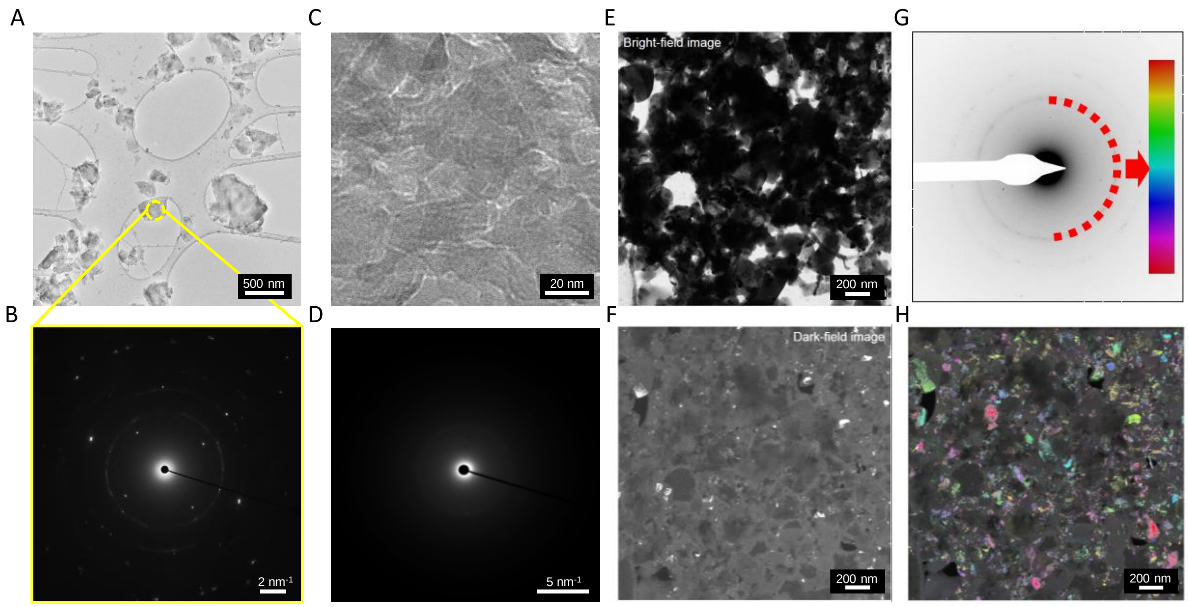

Figure S1: TEM characterisation of MgB_2 nanosheets. A) Bright-field TEM overview image of drop cast MgB_2 nano-platelets. A distribution of different sizes is observed. Thicker particles can be identified from lower contrast. **B)** SAED pattern on agglomerated particles measured in the region indicated by the yellow circle. The reflexes indicate high crystallinity of the particles in the probed region. **C)** TEM bright field image on particles left in the supernatant after centrifugation. While few thicker particles can be identified, mostly very small (~20-50 nm) and thin structures are left behind in the supernatant. **D)** SAED pattern corresponding to particles shown in C. Based on their blurred-out appearance, geometry and intensity of the reflexes, particles in the supernatant can be considered amorphous. **E-F)** TEM overview image on a Langmuir-type deposited sample in bright field (E), and in dark field mode (F). The network-like structure of the individual nanosheets in the deposited film can be identified in dark field mode. **G)** SAED measured across the deposited film (shown in E-F). The ring-like pattern indicates no preferential orientation of nanosheets in the network. **H)** False colour dark field image for the nanosheet orientation based on SAED reflexes as indicated by the colour scale in (G). The color-coded image was created from multiple dark-field TEM images corresponding to different orientations from the marked ring of Bragg reflections in the SAED pattern. As already indicated by the ring-like pattern, no preferred orientation of nanosheets within the network can be identified.

2.2 AFM Measurements

For accurate determination of the nanosheet dimensions after centrifugation, statistical AFM analysis was performed on drop-cast nanosheet samples. The evaluation includes manual measurement of the lateral sheet length (L) and width (W, with $L > W$ and $L \perp W$) and thickness (T) of >1500 individual nanosheets from the stock-like dispersion. The measured lateral dimensions were corrected for cantilever broadening and pixilation effects by calibration via transmission electron microscopy (TEM), as discussed in more detail in previous work.[1] For better comparability to other 2D materials, the measured thickness was converted into the nanosheet layer number (N) facilitating step height analysis as reported previously.[5-8] In brief, the measured height is influenced by the chemical characteristics of the surface and the measuring parameters (*e.g.*, measurement set point, free amplitude, *etc.*).[5-8] Additionally, it is important to account for solvent residues, which can adhere to the nanosheet surface or can be trapped in between the sheet and the substrate. Hence, the measured height from the AFM analysis on LPE nanosheets gives an apparent height, which is proportional but not equal to the true height.[9] To convert this apparent height into the nanosheet layer number, step height analysis can be performed on incompletely exfoliated nanosheets. In this approach, line profiles are extracted from steps and terraces of nanosheets, as previously demonstrated for TMDs,[1] $Ni_2P_2S_6$,[10] $RuCl_3$,[11] and $CrTe_3$[12] nanosheets. Statistical analysis of such steps heights reveals discrete steps of 0.8 nm for MgB_2 nanosheets, which is used to convert the apparent height measured in AFM to the number of layers (see Figure 3D-E in the main manuscript).

We note that statistically determining the dimensions of nanosheets in size-selected fractions enables a comparative analysis of the average nanosheet morphology across various materials. This is crucial for evaluating the exfoliation process, specifically the ease and quality of exfoliation. The current understanding of the processes associated with liquid-phase exfoliation suggests that it is primarily influenced by the ratio of in-plane to out-of-plane binding strength as previously discussed.[3] Several methods exist to quantify this parameter. One method involves calculating the arithmetic mean length-to-thickness aspect ratio. Another approach, as suggested in the literature,[13] involves analysing the natural logarithm of the aspect ratio, which typically follows a Gaussian distribution, allowing for reliable fitting to determine the average. Additionally, recent developments have introduced topological vectors to describe nanosheet morphology and shape[14]. However, these methods require large datasets, often comprising tens of thousands of nanosheets, which are not available for the air-sensitive MgB_2 nanosheets examined in this work.

In earlier work, we used a plot of fraction-averaged length as a function of thickness, which followed a power law, to extrapolate the characteristic monolayer length.[3] This method, however, can be affected by subtle differences in centrifugation-based size selection. In this study, we adopt a different approach to eliminate the influence of centrifugation while still enabling a reasonable comparison with published data on other materials. We first aggregate all data points on nanosheet length (or area, estimated as the product of length and width) and the corresponding thickness. This large dataset represents the accessible nanosheet lateral sizes and thicknesses producible by sonication-assisted liquid-phase exfoliation and accessible by centrifugation-based size selection within the 200–10,000 *g* range used for the preparation of metal diboride nanosheets in this work, making it qualitatively representative of the nanosheets in the as-prepared stock dispersion. We note that the centrifugation is necessary to remove unexfoliated material a low centrifugal acceleration, and to concentrate the nanomaterial in a second step at high speed. This also allows to remove impurities and defective material, which remain in the supernatant.

This dataset is then grouped into bins of a certain thickness range, ensuring that each bin contains at least 70 nanosheets. In each bin, the arithmetic average nanosheet area is calculated and plotted against the nanosheet layer number, as shown in Figure S2. Similar to the fraction-averaged data, a power-law relationship between area and the number of layers is observed, which can be fitted to an empirical equation of the form[3] $\langle LW\rangle = {D_{ML}}^2\langle N\rangle^{\beta}$, where D_{ML} represents the characteristic lateral nanosheet size associated with monolayers. This approach is comparable to, but not identical to, the method we previously used to determine the characteristic monolayer length through extrapolation.[3] The main difference between the two methods lies in the proportionality between the nanosheet area and layer number, indicated by the exponent (β in the equation above), which differs between fraction-averaged ($\beta \approx 2$),[3] and N-averaged data ($\beta \approx 1.2$).[15] Further experimental and theoretical work is required to fully understand this discrepancy.

The key outcome of this analysis is a reliable comparison of the ease of exfoliation of MgB_2 in comparison to other materials such as graphene,[3] $PtSe_2$,[16] and layered hydroxides, such as $Mg(OH)_2$,[17] and $Co(OH)_2$,[18] which we re-analyzed from our previously published work as reference points. As demonstrated in Figure S2, the MgB_2 nanosheets exhibit a similar quantitative relationship between area and layer number as $PtSe_2$, implying that the ratio of in-plane to out-of-plane binding strengths is comparable. Overall, the nanosheets are laterally smaller at a given layer number than graphene by approximately an order of magnitude.

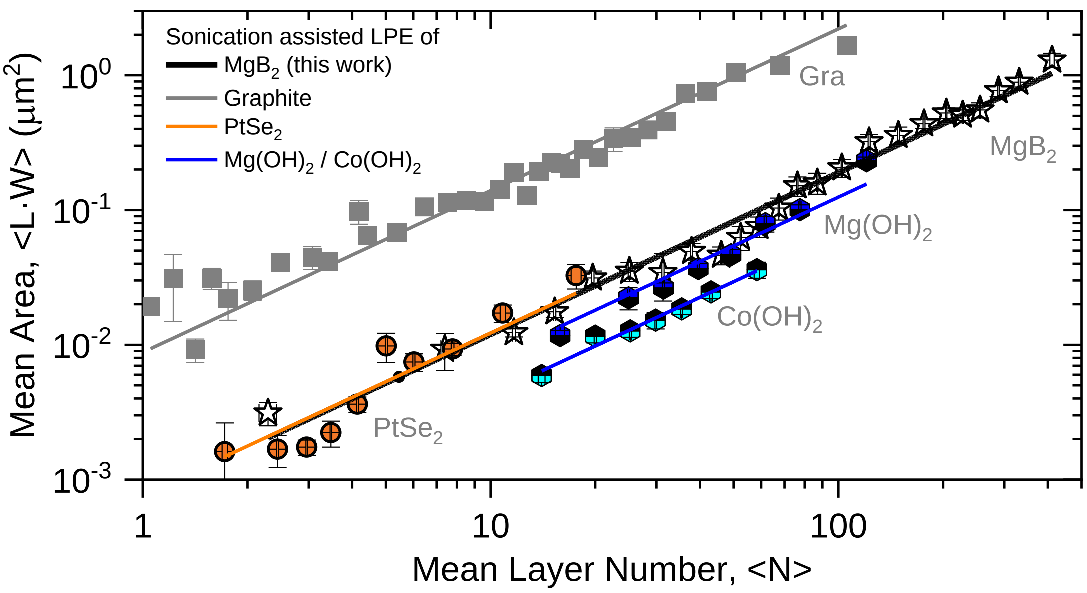

Figure S2: MgB_2 nanosheet dimensions compared to other materials. Plot of the arithmetic mean nanosheet area over the mean nanosheet layer number. The MgB_2 nanosheet area follows a power law increasing with the nanosheet layer number as observed for other materials in previous work.[3, 15] Additional data on LPE graphite,[3] $PtSe_2$,[16] $Mg(OH)_2$[17] and $Co(OH)_2$[18] is shown for comparison. Adapted with permission from C. Backes, et al., ACS Nano 2019 13 (6), 7050-7061, DOI: 10.1021/acsnano.9b02234. Copyright 2019 American Chemical Society.

3 Network Characteristics for Different Deposition Methods

Following the successful preparation of nanosheets with confirmed properties via EDX and IR spectroscopy, these nanosheets were processed into thin films to investigate their electrical conductivity. This study employed three distinct deposition techniques: vacuum filtration, spray coating, and a modified Langmuir Schaefer deposition. This approach enables the examination of how film morphology, influenced by the specific deposition methods, impacts both the electrical properties[19] and the spectroscopic response of the materials (*i.e.*, potential signs of material decomposition).

To achieve this, the same spectroscopic methods utilized for the initial nanosheet characterization described earlier were applied to the deposited nanomaterial. These methods facilitate a combination of optical and electron microscopy, EDX and IR spectroscopy, as well as I/V measurements (Figure S3) to create a complete picture of the impact of processing conditions on the properties of the nanosheet network. These preliminary results were used to identify the most promising method for MgB_2 nanosheet thin film fabrication and for subsequent optimisation.

Significant differences in film morphology are evident from optical microscopy (Figure S3A-C). Vacuum filtration results in films with noticeable thickness variations and

structural voids. Notably, the material appears to easily detach from the membrane, as indicated in the inset of Figure S3A. In direct comparison, sprayed films exhibit a greater degree of homogeneity (Figure S3B). However, periodic variations in height are observable, evidenced by contrast changes across the film. This phenomenon could be attributed to the choice of room temperature for drying, aimed at preventing temperature-induced nanosheet oxidation. The Langmuir-type deposited thin film displays islands of varying nanosheet coverage density (Figure S3C), a feature later identified as resulting from insufficient material injection. During the drying process, areas with densely packed tiled nanosheets form, leaving regions with sparser coverage when the injection volume is inadequate.

SEM images of the deposited MgB_2 films reveal distinct differences among the three deposition methods. In the case of vacuum filtration, the images depict aggregates and bulges of nanoparticles (Figure S3D). This suggests that the vacuum filtration process results in the clustering of MgB_2 particles, leading to uneven film morphology.

In contrast, the sprayed films display a more uniform and homogenous coverage (Figure S3E). Here, the material appears evenly distributed across the substrate, resulting in a smoother and more consistent film structure.

The Langmuir-type deposition showcases a unique morphology, with a single layer of tiled MgB_2 nanosheets sparsely covering the substrate (Figure S3F). The nanosheets are arranged in a mosaic-like pattern, leaving areas of the substrate exposed. This distinct feature is attributed to the precise control over nanosheet alignment and packing density achieved through Langmuir deposition. Note that the packing of nanosheets deposited by Langmuir deposition may be further controlled by varying the injection volume during the process, allowing for fine-tuning of the film morphology.

The EDX analysis on all three films shows significant differences for the EDX suggested oxygen content of the individual deposition methods (Figure S3G-I). The vacuum-filtrated film reveals significant oxidation, indicated by an EDX-suggested ratio of Mg:B:O at approximately 1:1.5:2.7 (Figure S3G). This substantial increase in oxygen content may partially be caused by the porous nature of the filtered films, where oxygen may be trapped. However, the more likely scenario is that the porosity leads to an increased material oxidation due to higher exposure of individual sheets to the environment.

For the sprayed film, oxidation is also observed, albeit to a lesser extent. The EDX data suggests a Mg:B:O ratio of approximately 1:2.8:1.1 for the sprayed nanosheet film (Figure S3H). This indicates that the film has undergone oxidation compared to

the initial nanomaterial, which exhibited a Mg:B:O ratio of 1:1.9:0.2 (after drop cast in an Argon atmosphere). We attribute the increase of the oxygen content to prolonged exposure to ambient oxygen during the process as low deposition rates were required for film fabrication at room temperature.

Conversely, the Langmuir-deposited film shows a lower oxide content. The EDX analysis yields an Mg:B:O ratio of approximately 1:2:0.8, suggesting a reduced presence of oxygen in the film (Figure S3I). We account the lower oxygen content in the Langmuir films to the established drying protocol in an Argon flow, which was not possible for filtered and spray coated films, where the nanomaterial was exposed to ambient conditions for the timeframe required for the deposition process.

In further analysis, IR transmittance was recorded for the deposited nanomaterial. For this purpose, nanomaterial was collected from the membrane and substrate surface in an inert atmosphere. The obtained spectra were compared to nanomaterial drop-cast under inert conditions (Figure S3J-L).

It is apparent from the spectra that the nanosheets undergo significant oxidation during the filtration process (Figure S3J). This result is somewhat unexpected since the filtration process exposes the samples to ambient conditions for only about 15 minutes. One possible explanation is the high porosity of the film, which could potentially trap oxygen in hollow spaces, leading to ongoing material oxidation even after transferring the samples to an inert atmosphere.

Spectra obtained from the sprayed nanosheets exhibit only slight indications of nanosheet decomposition (Figure S3K). However, it is important to note that the amount of material that could be scraped off the substrate was significantly lower compared to the filtered nanosheets. Consequently, the quantity of material available for IR measurements may have been insufficient for drawing meaningful conclusions. Additionally, the overall signal intensity of the initial material is poor. Nonetheless, faint indications of ν OH-stretch modes and δ B-OH vibrations can be observed in the spectra of the sprayed material, as denoted by the dashed blue lines in Figure S3K.

For IR spectroscopy on Langmuir-deposited nanosheets, material from previous depositions with poorly covered films was combined to achieve a sufficient material mass for measurements. While the spectra do show additional modes corresponding to oxidized boron species (Figure S3L), unlike the sprayed films, no quantitative information can be derived from IR spectroscopy. It's important to consider that comparing the spectra of Langmuir Schaefer-type deposited films to the sprayed films may be misleading due to the limited material accessible for IR measurements after the spraying process, as discussed above.

Finally, I/V characteristics of each film were investigated using silver-painted electrodes. Although we anticipated low conductivity in partially oxidized and porous films, the results provide valuable insights into charge transport. These insights can be compared across different processing techniques, which yield varying degrees of oxidation, porosity, and film morphology.

The measured I/V curves for the filtered nanomaterial (Figure S3M) indicate non-ohmic transport behavior within the measured voltage range of ±1.5 V. However, considering the network's porosity, the current is relatively high. The film's extracted conductivity around the origin (±100 mV) was determined to be 2.24 $S \cdot m^{-1}$.

In contrast, the current-voltage characteristics recorded for the sprayed nanosheet film were acquired in a potential range between ±8 V (Figure S3N). The comparatively higher voltage was required to study the charge transport characteristics of the sprayed nanosheet film compared to the filtered nanosheets due to a plateau in the I/V curve between ±5 V. The curvature in the I/V measurements implies non-ohmic transport behaviour for the sprayed films as well. After crossing the "potential barrier," currents in the microampere range were measured within the applied potential range. Although the conductivity around the origin appears nearly constant, ohmic conductivity can be fitted to Ohm's law at low potentials (±500 mV, see inset Figure S3N). The extracted conductivity in this voltage range was determined to be $1.06 \cdot 10^{-2}$ $S \cdot m^{-1}$. The curvature in the graph resembles a Schottky barrier, although further analysis was not conducted as additional deposition techniques were in focus of this study.

At last, the I/V characteristics of the Langmuir-type deposited film were studied (Figure S3O). The measurements revealed high ohmic conductivity within the measurable range. Linear fitting of the data suggests a conductivity of $2.25 \cdot 10^5$ $S \cdot m^{-1}$. This is surprising, considering that the substrate was not entirely covered over the entire area. It suggests that only a few conductive pathways of tiled nanosheet networks already lead to benchmark conductivities. For comparison, typical conductivities of sprayed graphene nanosheets show a conductivity of $\sim 3 \cdot 10^3$ S/m[20] (sprayed MgB_2: $1.06 \cdot 10^{-2}$ $S \cdot m^{-1}$, likely due to severe oxidation, as discussed above).

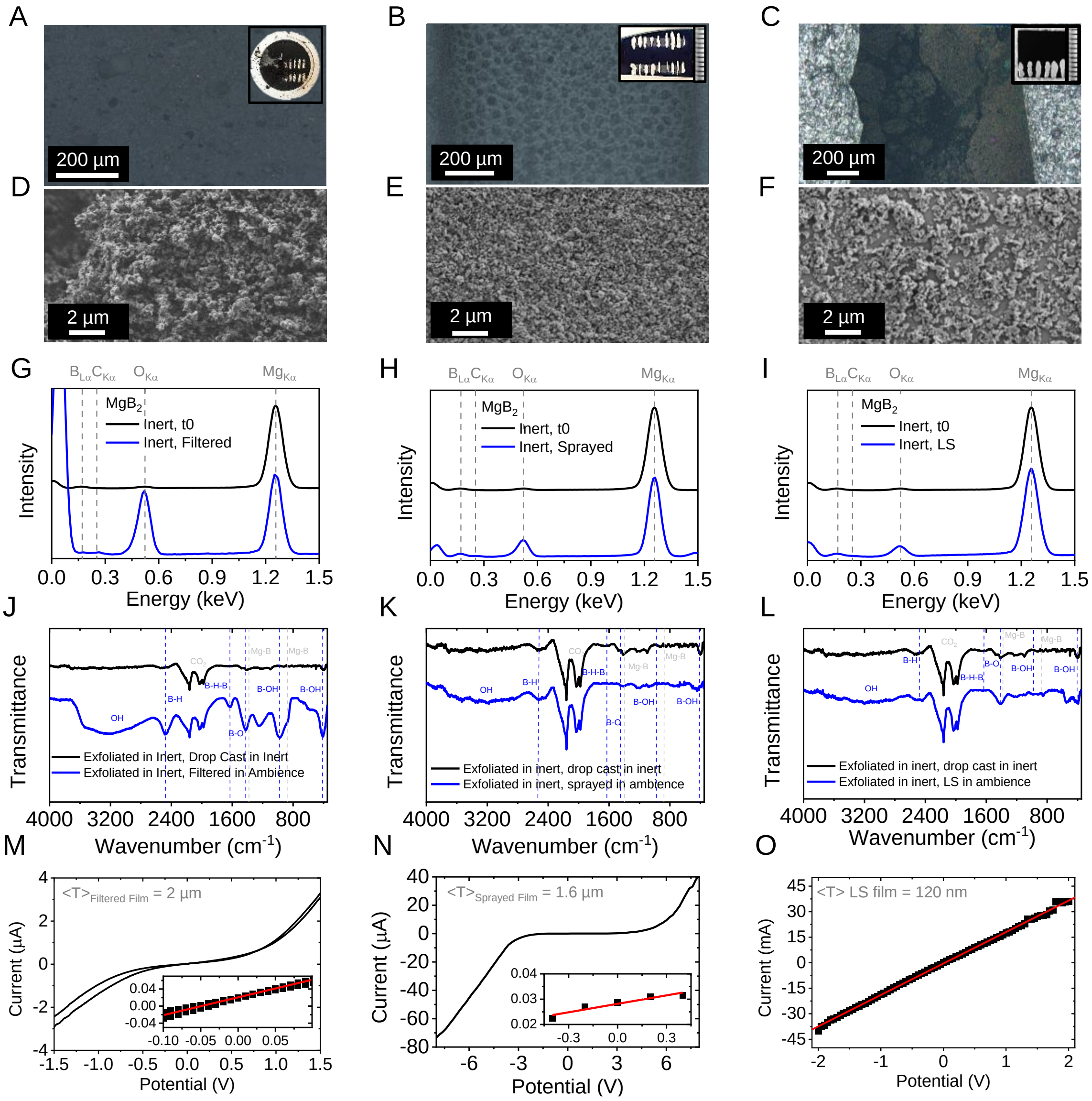

Figure S3: Impact of deposition technique on nanosheet thin film characteristics. A-C) Optical microscopy image of deposited MgB_2 nanosheet films facilitating vacuum filtration (A), spray coating (B) and a Langmuir-type deposition (C). **D-F)** SEM measurements on the same films as shown in A-C. The measurements show significant changes for the morphology of nanosheets deposited by vacuum filtration (D), where nanosheets seem to form aggregates, spray coating (E), which results in homogenous coverage in the nanoscale, and Langmuir-type deposition (F), for which a monolayer of the nanomaterial forms a sparsely covered network across the substrate. **G-I)** EDX measurements on deposited nanomaterial. Significant oxide contributions are observed for nanosheets process from all three deposition techniques. An Mg:B:O ratio of 1:1.5:2.7 is suggested for the vacuum filtrated material (G), for the spray coated material (H), a ratio of 1:2.8:1.1 is found, and for the Langmuir-type deposited nanomaterial (I), a ratio of 1:2:0.8 is found according to EDX measurements. **J-L)** IR transmission measurements on deposited nanosheets. While significant changes to the nanomaterial are observed after vacuum filtration (J), only subtle indications material decomposition are observed for sprayed (K) and Langmuir-deposited nanomaterial (L). **M-O)** I/V characteristics for the three deposition techniques using painted silver electrodes. While these results are considered preliminary as the electrode area is poorly defined, significant differences are observed for all three deposition techniques. The Langmuir-type deposited network is the only example demonstrating ohmic conductivity.

The I-V characteristics of three distinct thin films (see Figure S3M-O) exhibit varying electrical behaviour. The Langmuir-type thin film show linear I-V curves, whereas the other two deposition techniques show noticeable curvature at higher potential. To confirm whether the behavior is Ohmic or non-Ohmic, the current density (J) is calculated using $J = \frac{I}{W \cdot \langle T \rangle}$, where W and $\langle T \rangle$ are the electrode length and average thickness, respectively. J is plotted against the electric field (E) in V m^{-1} (Figure S4A), with E being estimated by dividing the applied potential by the channel length (*i.e.*, the distance between electrodes).

In the Langmuir-type deposited film, a linear J-E behaviour (*i.e.*, $J = \sigma E$) persists for the entire field range, suggesting semi-metallic properties of the nanosheets. In contrast, non-linear behavior is observed for the filtered, and the spray deposited films, which implies that the nanosheets lose their semi-metallic properties and potentially undergo oxidation, leading to semiconductor-like transport characteristics. Consequently, Schottky barriers are formed at the metal/semiconductor interface (*i.e.*, oxidized MgB_2/Ag), resulting in non-linear I-V curves.

At relatively low field (or potential), the transport of carriers is hindered by the potential barrier at the interface, leading to low current density. As E increases, the potential barrier height is compensated by the applied potential, enabling a greater number of carriers to transport across the interface and resulting in an exponential increase of J. Nevertheless, the current densities for the filtered and sprayed thin films at any potential are significantly lower than those for the Langmuir-type film. This implies that the conductivity of the latter is higher than that of the other deposition techniques, highlighting the advantage of the adapted Langmuir-Schaefer method in preserving the pristine properties of the nanosheets.

In Figure S4B, a systematic change in the I-V curves over time in ambient conditions is observed. Although initially, ohmic behaviour is obtained, curvatures become apparent over time after samples are exposed to air. We attribute this behavior to a gradual oxidation of nanosheets in ambient air. When left exposed to the ambient environment, some nanosheets, or a portion of each individual nanosheet, may retain their semi-metallic properties, while the remainder or the remaining part of the nanosheets transforms into a semiconducting type over time. The semiconducting nanosheets develop a Schottky barrier at the metal/semiconductor interface, causing non-linear trends in the I-V characteristics. It can be anticipated that with more extended oxidation over time for the Langmuir-type thin films, their I-V curves will eventually exhibit more pronounced curvatures, similar to those observed for the filtered and sprayed thin films. We note that for these experiments were performed prior to optimization and with the use of painted silver electrodes (see inset of Figure S3A-C for examples). While the conductivity is lower than for optimised deposition protocols,

which are described in detail in the methods section, the results demonstrate the potential of the Langmuir-type deposition, and the necessity for inert sample handling. Later optimization allowed to significantly improve the conductivity.

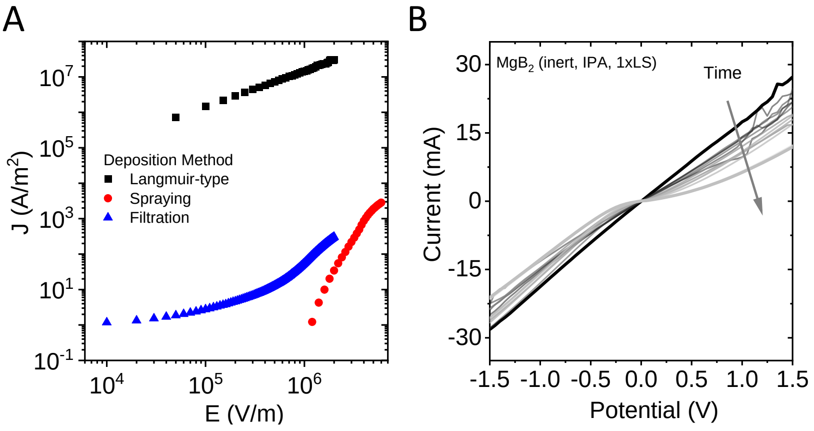

Figure S4: Morphology and time dependent charge transport behaviour of MgB_2 nanosheets. (A) Plot of current density (J) versus electric field (E) for the filtered, sprayed and Langmuir-type MgB_2 nanosheet films. **(B)** Change of the I-V characteristics for the Langmuir-type film over time left in ambient environment.

4 Layer by Layer Deposition of Tiled Nanosheet Networks

In the perspective of increased conductivity after deposition of multiple layers of MgB_2 nanosheets, consecutive depositions were performed on the same substrate. Although some material may be removed when moving a previously deposited film through the water surface due to the surface tension, it was feasible to deposit multiple layers using the Langmuir-Schaefer technique, resulting in increased thicknesses. We note that employing extended drying times and annealing in an inert atmosphere is important as it improves the adhesion of the nanomaterial in the film. SEM images were captured at different magnification of the same region of the nanosheet network after four consecutive iterations of Langmuir-Schaefer deposition (Figure S5). It is evident that nanosheet alignment has improved compared to the initial films, and no apparent aggregation is observed. The film appears cohesive and exhibits homogeneous morphology with an average thickness of approximately 538±35 nm, as determined by profilometry at various locations on the substrate. The SEM images suggest that individual nanosheets are well-aligned on the substrate surface.

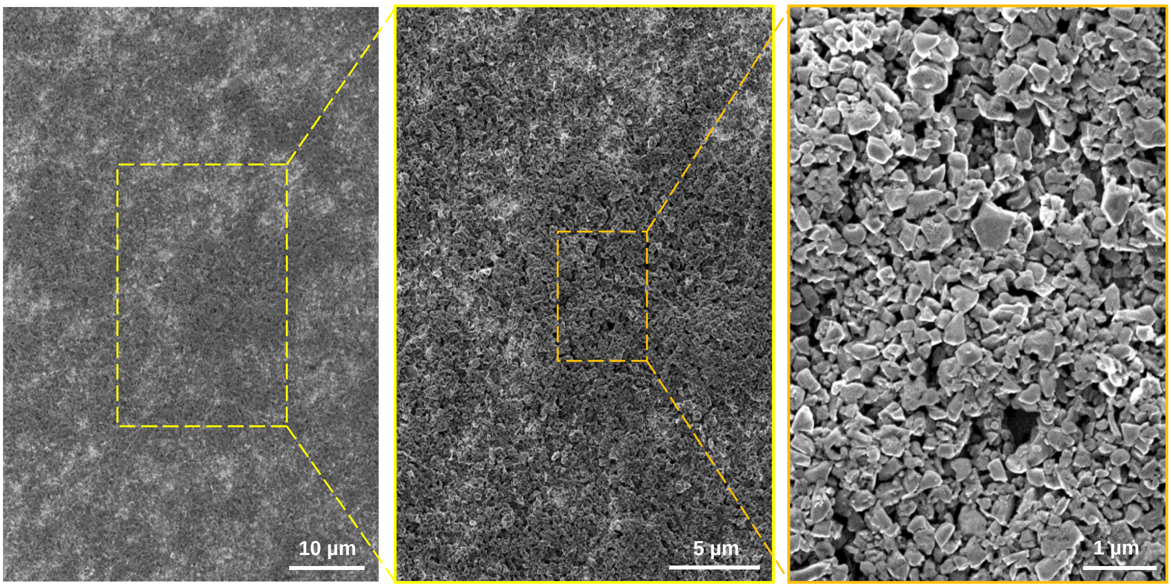

Figure S5: SEM images of a Langmuir Schaefer-type deposited nanosheet network of MgB_2 nanosheets at different magnification. The film was made by four subsequent Langmuir-type depositions of MgB_2 nanosheets on the same substrate. Individual sheets are clearly resolved and show homogenous coverage of the substrate surface.

In further analysis of the impact of thickness on the charge transport characteristics across multiple iterations of Langmuir-type depositions of MgB_2 nanosheets, we performed I/V and profilometry measurements (Figure S6). These experiments were carried out on films subjected to 1, 2, and 4 iterations of the Langmuir-type deposition process (Figure S6A). Ohmic transport properties were consistently observed for all deposition numbers (Figure S6A-B).

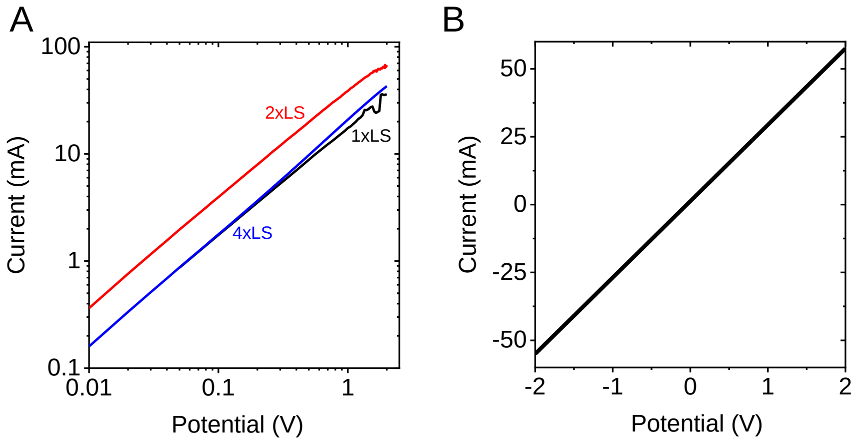

Figure S6: Thickness dependence on electrical performance of Langmuir-type deposited MgB_2 nanosheet films. (A) I/V measurements for 1, 2 and 4 subsequent depositions of MgB_2 nanosheets using the Langmuir-Schaefer technique on a logarithmic scale. For all deposition numbers, a similar conductance is observed. **(B)** I/V characteristics of the four-times deposited MgB_2 film shown in Figure S5.

5 Exfoliation and Langmuir-Type Deposition of CrB_2 and ZrB_2

To assess if results can be transferred to similar material systems, CrB_2 and ZrB_2 have been exfoliated and deposited similar to MgB_2 for a direct comparison. To this end, both compounds have been exfoliated using bath sonication under ambient and inert condition, respectively. The normalized absorbance spectra for all materials are presented in Figure S7A-C, illustrating a side-by-side comparison for exfoliation under inert (black trace) and ambient (red trace) conditions. In each case, alterations to the spectral profile in the UV-region are observed, which is indicative of nanomaterial oxidation when exfoliated in air. Consequently, all subsequent experiments were conducted under inert conditions.

To estimate the mass of the dispersed nanomaterial, the optical coefficients were determined using vacuum filtration. This allows to calculate the coefficient spectra for absorbance (α), extinction (ε), and scattering (σ) of each nanomaterial (Figure S7D-F).

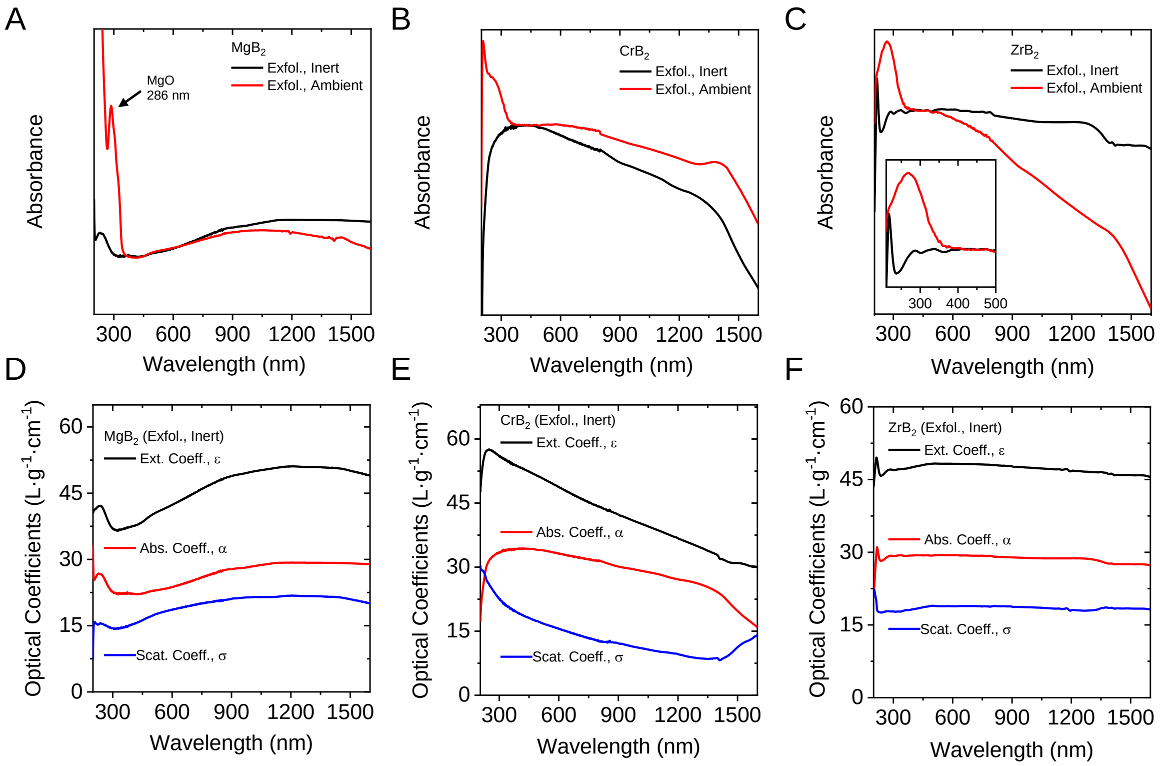

Figure S7: UV-Vis measurements for different metal diboride nanosheets in IPA. (A-C) Normalised absorbance spectra of exfoliated MgB_2 (A), CrB_2 (B) and ZrB_2 (C). The exfoliation was tested under inert (black) and ambient (red) conditions for each material to test for signs of oxidation when in contact with air. **(D-F)** Extinction (ε, black), absorbance (α, red) and scattering (σ, blue) coefficient spectra for exfoliated MgB_2 (D), CrB_2 (E) and ZrB_2 (F).

Nanomaterial from both additional materials was deposited using the modified Langmuir Schaefer method, employing conditions similar to those previously utilized

for MgB_2 nanosheets deposition. The film morphologies after four iterations of deposition are depicted in Figure S8 (for CrB_2) and Figure S9 (for ZrB_2), respectively.

For CrB_2, optical microscopy indicates homogeneous film morphology across the entire substrate area (Figure S8A). However, at higher magnification, local imperfections in the tiled nanosheet alignment are apparent, as observed by SEM (Figure S8B). Although spots with pinholes were observed at various positions on the substrate, the alignment of nanosheets into a tiled network is evident from microscopy. While operator parameters may contribute to this deviation, the oxidation of the nanomaterial could also impact film formation, as significant oxidation of the material is evident from EDX spectroscopy, even for nanomaterial deposited immediately after preparation in an inert atmosphere.

EDX spectroscopy was conducted on the nanomaterial drop-cast under inert conditions immediately after preparation and after Langmuir-Schaefer deposition. The measurements suggest significant oxidation of the material after Langmuir-type deposition (EDX stoichiometries (Cr:B:O) are 1:2.4:0.2 for drop-cast and 1:2:0.6 for Langmuir-Schaefer deposited nanosheets).

Additionally, I/V characteristics were measured on a Langmuir-deposited nanosheet film after four iterations of deposition (Figure S8D). Indications of material oxidation are evident from the curvature in the I/V plot. As the nanosheets do not exhibit ohmic charge transport behaviour within the measured potential range of ±1 V, the film's conductivity was determined by linear fitting within a small voltage range with a linear I/V dependence (±100 mV), as indicated by the red line in the inset of Figure S8D. The fit suggests an initial conductivity of the CrB_2 nanosheet film of $1.06 \cdot 10^2$ $S \cdot m^{-1}$. We note that for CrB_2 rectifying transport behavior is observed. While the reasons for this cannot be elucidated without further experiments, a possible explanation could be that reactions with water occur for CrB_2 nanosheets. In future experiments, it will be important to find possible alternatives to water or protic solvents compatible with the film formation at the liquid/liquid interface, and to make to the process compatible with protocols suitable for deposition in an inert atmosphere. However, this is not the focus of this work.

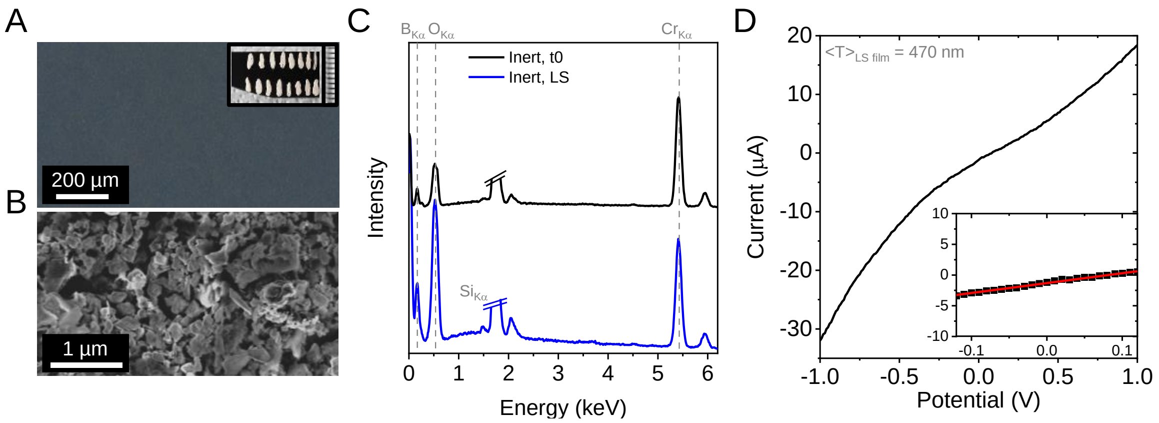

Figure S8: Thin film processed CrB_2 nanosheets by modified Langmuir Schaefer deposition. (A) Optical bright field image of the CrB_2 film. **(B)** SEM image of the CrB_2 film after four subsequent iterations of the Langmuir Schaefer deposition. The nanosheet film shows pin holes but overall complete coverage of the substrate with spot-to-spot variations on the morphology. **(C)** EDX spectra of pristine nanomaterial (black, drop cast in inert atmosphere) compared to Langmuir Schaefer deposited nanosheets (blue). Oxidation of the nanosheets is observed for the Langmuir-type deposited nanosheets, as well as for the reference. The EDX suggested stoichiometries are 1:2.4:0.2 and 1:2:0.6 (Cr:B:O) for the pristine and processed nanosheets, respectively. **(D)** I/V curve corresponding to the Langmuir Schaefer deposited nanosheets after four depositions. The sheets show deviations form ohmic behaviour, in line with material oxidation. The conductivity extracted within the linear I/V range of ±100 mV is $1.06 \cdot 10^2$ S·m^{-1}

For ZrB_2 nanosheet films produced after four consecutive Langmuir-Schaefer depositions, no noticeable inhomogeneities are apparent on the macroscale, as observed through optical microscopy (Figure S9A), or on the mesoscale, as seen in SEM imaging (Figure S9B). Moreover, EDX spectroscopy suggests minimal oxidation of the nanomaterial post-deposition, with nearly no deviation from the reference measurement on a drop-cast film in an inert atmosphere (EDX suggested stoichiometries (Zr:B:O) are 1:1.6:0.2 and 1:2:0.3 for drop cast and Langmuir Schaefer film, respectively, Figure S9C). In comparison to the results for MgB_2 and CrB_2, ZrB_2 appears to be less susceptible to oxidation. This observation is further substantiated by the ohmic behaviour in the I/V characteristics of the nanosheet network, as illustrated in Figure S9D. Fitting yields a conductivity of $3.95 \cdot 10^5$ S·m^{-1}.

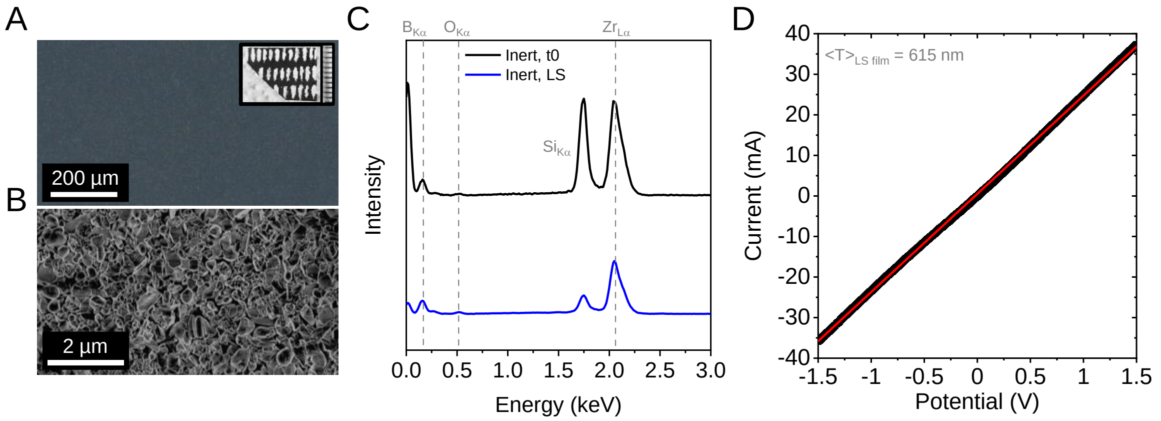

Figure S9: Thin film processed ZrB_2 nanosheets by modified Langmuir Schaefer deposition. (A) Optical bright field image of the ZrB_2 film. **(B)** SEM image of the ZrB_2 film after four subsequent iterations of the Langmuir Schaefer deposition. **(C)** EDX spectra of pristine nanomaterial (black, drop cast in inert atmosphere) compared to Langmuir-Schaefer deposited nanosheets (blue). Minute oxidation of the nanosheets is observed for the Langmuir-type deposited nanosheets, as well as for the reference. The EDX suggested stoichiometries are 1:1.6:0.2 and 1:2:0.3 (Zr:B:O)for the pristine and processed nanosheets, respectively. (D) I/V curve corresponding to the Langmuir Schaefer deposited nanosheets after four depositions. The sheets show ohmic behaviour, suggesting that no significant oxidation of the nanomaterial had occurred, in line with the EDX spectra. The conductivity extracted within the measurable range is $3.95 \cdot 10^5$ S·m^{-1}.

For both CrB_2 and ZrB_2, successful nanosheet deposition using our adapted Langmuir-type technique was achieved, and the data for both materials suggest similar results as observed for MgB_2 nanosheets. Overall, Langmuir-Schaefer deposition results in homogeneous coverage of the entire substrate area with minimized material oxidation, primarily exhibiting ohmic I/V characteristics. Such favourable results were not attainable with spray coating or filtration of the nanomaterial, which led to inhomogeneous coverage, adhesion issues, and severe oxidation. Hence, the Langmuir-Schaefer method emerges as the most promising among the three tested approaches. However, it should be noted that no systematic optimization was performed for either deposition method.

In further experiments, the nanosheet dispersions and the deposited Langmuir-Schaefer films were used for time-dependent Extinction measurements, and for the latter, additional time-dependent conductivity measurements were performed.

Time-dependent extinction measurements for nanosheet dispersions of all three materials are shown in Figure S10A (from left to right, MgB_2, CrB_2, and ZrB_2). For all three materials, systematic changes are observed over time, generally marked by an increase in the relative optical density in the UV-region, attributed to the formation of wide-bandgap metal oxides. While the spectral profile of each material remains largely unaffected above 400 nm, the overall optical density of the material decreases over time, aligning with a reduced contribution of metallic (or low-bandgap) material to the spectra and the formation of oxides.

In a direct comparison with nanosheet dispersions, the respective nanosheet films of MgB_2, CrB_2, and ZrB_2 after four iterative Langmuir-Schaefer depositions are displayed below (Figure S10B). Unlike the dispersions, the behaviour of the deposited nanosheets varies for each material. While for MgB_2 (left), noticeable changes in the

spectral profile and a decrease in optical density are observed. There is no apparent change in the photo-spectroscopic response of CrB_2 (centre) over time. This is surprising since the spectra of the deposited material resemble the spectral profile of the initial nanomaterial in dispersion. However, a peak is observed in the UV region of the deposited film, indicating material oxidation. One plausible explanation could be that the material oxidized significantly between the start of the deposition steps and the first measurement. While this aligns with the general air sensitivity of CrB_2, as discussed above, it remains unclear why no changes in the spectra are observed over time. Finally, deposited ZrB_2 nanosheets (right) exhibit systematic changes in the spectral profile in line with the nanomaterial in dispersion: the initial peak in the UV region red-shifts over time while the overall optical density decreases.

6 Decomposition Kinetics for MgB_2, CrB_2 and ZrB_2

For a more quantitative discussion of the observed trends for both dispersed and deposited nanosheets, they were subjected to a kinetic analysis (Figure S10C) for samples stored under light exclusion in air (dispersions were stored in 4 ml clear screw-cap vials, films in PS plastic boxes). To achieve this, specific wavelengths purely assigned to the respective MB_2 nanomaterial, where no change in the spectral profile but a systematic decrease in optical density is observed, were selected. These respective wavelengths are proportional to the change in material concentration (*e.g.*, O.D.1000 nm ∝ [MgB_2]) and provide information about the rate of material consumption upon degradation. Optical densities at these wavelengths were plotted as a function of time for all materials. The discussion focuses on the most systematically behaved wavelength for both the nanosheet dispersion and the film, respectively, being 1000 nm in the case of MgB_2 and CrB_2 and 600 nm in the case of ZrB_2 (Figure S10C, upper row). In all cases, a reasonable agreement with a single exponential decay is found, which is used to extract the portion of reacted material and the material's half-life ($t_{1/2}$). The results of the fit are summarized in Table 1 in the main MS and discussed below, in comparison to trends extracted from time-dependent electric measurements.

However, in a further analysis of the extinction spectra, an attempt was made to extract the rate law for each material. To achieve this, the natural logarithm and the inverse of the optical density change were plotted as a function of time (Figure S10C, centre + bottom row). It is noteworthy that the linearization of the data in either of the plots implies behaviour in agreement with a first or second-order rate law, respectively.

The data is reasonably fitable to a linear trend for all dispersions in both plots, while a roughly linear trend is only evident when plotting the inverse of the optical density for the films of MgB_2 and ZrB_2. While a first-order rate law would be consistent with diffusion-limited degradation of the material in the dispersion, a second-order rate law would make sense for the nanosheet films as the nanosheet surface is exposed to ambience directly. However, the linearization for both the plot of the natural logarithm and the inverse optical density of the data acquired for the dispersion renders the kinetic analysis from extinction spectroscopy alone inconclusive. The trends imply that either the time range in which the measurements are taken or the absolute range in which the optical density changes are too small for a meaningful conclusion on the rate law of the decomposition.

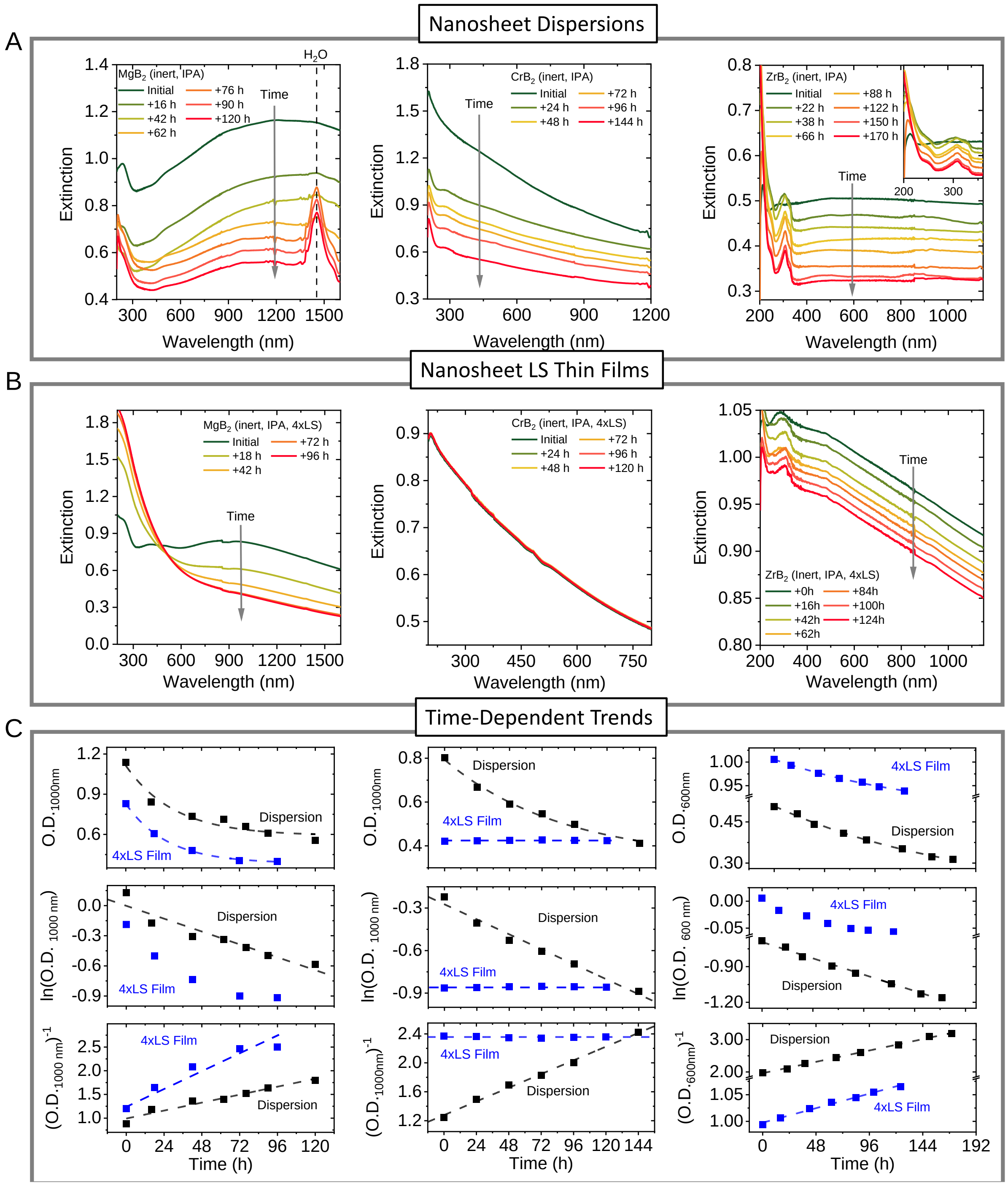

Figure S10: Time-dependent UV-Vis measurements of MgB_2 (left), CrB_2 (centre) and ZrB_2 (right) nanosheets in ambient conditions. (A) Extinction spectra of the same MB_2 nanosheet dispersion in IPA acquired over time. The spectra show systematic changes of the optical profile for all three materials under study. **(B)** Time-dependent extinction spectra of four times deposited MB_2 nanosheet networks made from the same dispersions as shown in (A). The optical profile changes systematically over time for MgB_2 (left) and ZrB_2 (right), similar to the nanosheets in dispersion, while CrB_2 (centre) shows no change over time. **(C)** Plots of the optical density change (upper row), the natural logarithm of the optical density (centre row), and the inverse optical density (lower row) to study reaction kinetics of the material decomposition. The decomposition of nanosheets is dispersion is expected to show a first order rate law, following an exponential trend of the optical density change. This is due to diffusion limited reaction conditions in the initially dry and deoxygenized solvent. The films are exposed to oxygen and ambient water and follow a second order rate law for MgB_2 and ZrB_2. Note, that the data acquired for the dispersion can be fit to the expected behaviour for first and second order rate law. The data for linearization in agreement with a first or second order rate law is therefore considered inconclusive.

To delve deeper into the decomposition mechanism of the deposited nanosheet films, I/V characteristics for nanosheet films were monitored over time for all three materials for two different films, being after a single deposition and after four subsequent depositions, respectively (Figure S11). In Figure S11A-C, the recorded I/V curves for a single Langmuir Schaefer deposition are shown. Similar to Figure S10, results for MgB_2, CrB_2, and ZrB_2 are arranged from left to right. The I/V curves for nanosheet films after four iterations of the Langmuir Schaefer deposition are displayed below in Figure S11D-F. For each material, the I-V curves suggest an increase in resistance over time. In all cases, the material undergoes a transition deviating from ohmic conduction over time, aligning with the observed material oxidation. We note that for the purpose of these measurements, painted silver electrodes were used. While the electrodes were deposited by hand as described in more detail in the methods section, absolute values are only indicative for the electrical behavior of the nanosheet network. However, relative trends on the change of I-V characteristics over time can be correlated to elucidate kinetic aspects on the material oxidation.

Given the consistent behavior observed for films after a single deposition and those after four iterations, and to enable a comparison to time dependent UV-Vis measurements, where four depositions were required for a reasonable signal to noise ratio, the change in conductivity over time for films produced after four depositions is correlated with the time-dependent extinction spectra of similar films after four depositions (Figure S10). While the data for MgB_2 and ZrB_2 shows a reasonable agreement between the two datasets from extinction spectroscopy and conductivity measurements, the dataset for CrB_2 exhibits a distinct behavior in its I/V characteristics compared to the extinction. This implies that the extinction measurement for the deposited CrB_2 nanosheets may not be reliable, as discussed further above. However, similar to UV-Vis measurements, changes to the I/V characteristics can be used to study the degradation kinetics. For this purpose, the network conductivity is fitted to a single exponential decay to independently extract the portion of reacted material and the half-life of the nanomaterial, in addition to the data from extinction spectroscopy (Figure S10G-I). The results of the evaluation are shown in Table 1 in the main MS.

The fits (Figure S11G-I) suggest that for all three materials, almost complete degradation is expected over time for deposited films, while the oxidation in dispersion seems to stop after roughly 50% of the initial material was consumed, as suggested by the time-dependent extinction spectroscopy. The material's half-life appears to vary more significantly than the portion of reacted material for the individual compounds.

While the data implies that half of the initial MgB_2 degrades within less than one day for both, deposited and dispersed nanosheets, CrB_2 shows a slightly slower degradation with approximately 2-3 days until 50% of the material is decomposed if stored

in dispersion, or about 1 day after the deposition into a thin film. ZrB_2 shows the longest half-life for more than 4 days independent of the storage in dispersion or after the deposition. However, it is important to keep in mind that the reported values are only suitable to give an impression on the materials' sensitivity to ambient conditions rather than a guaranteed timeframe for the material processing and encapsulation. Absolute values may differ even for slightly different storage conditions (*e.g.*, light exposure, humidity, room temperature, material's size distribution). For a quantitative discussion of absolute values, trends for changes of each potential influencing parameter have to be isolated and systematically varied. However, such studies were not possible within the scope of this work.

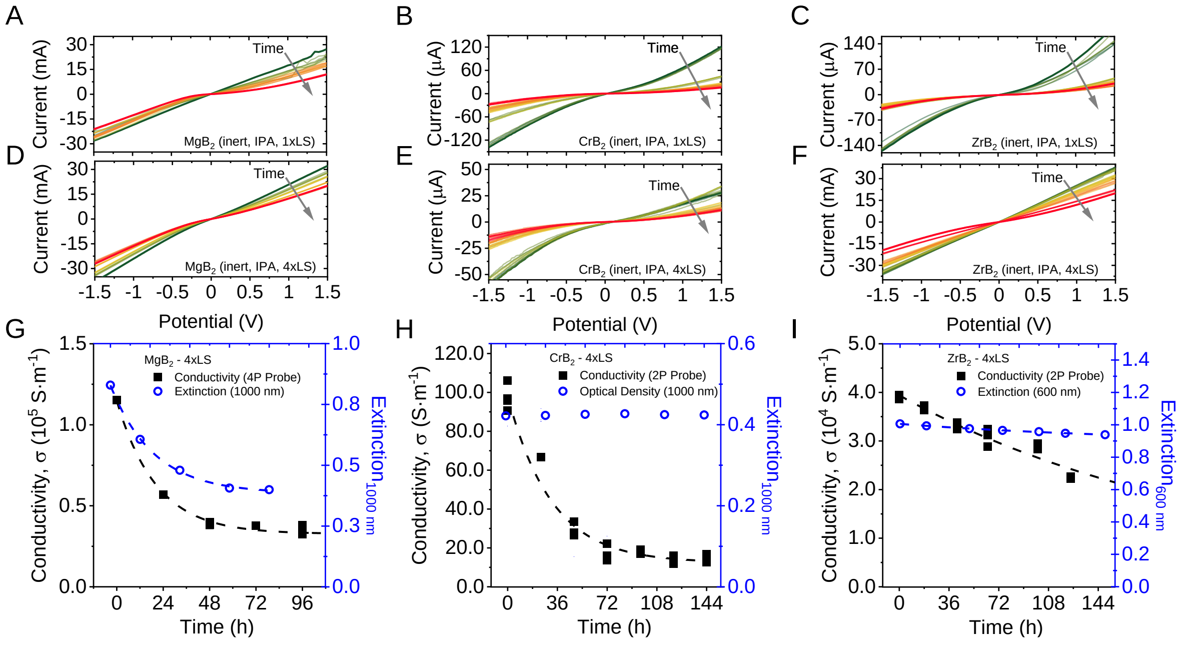

Figure S11: Degradation kinetics in metal diboride films from conductivity data and photospectroscopic measurements. (A-F) Change in the I-V response of Langmuir Schaefer deposited MB_2 nanosheet films as a function of time for a single (A-C) and four sequential (D-F) depositions. The I-V curves in (A, D) represent MgB_2 after one and four depositions, (B, E) represents CrB_2 after one and four depositions, and (C, F) represents ZrB_2 after one and four depositions. In all cases the measured current drops with exposure time to ambient conditions. (G-I) Change in the network conductivity and optical density (at wavelengths attributed exclusively to each respective MB_2 material) as a function of time for MgB_2 (G), CrB_2 (H) and ZrB_2 (I). The films for each material were deposited using four sequential Langmuir Schaefer depositions. Both datasets follow a single exponential decay, which allows the portion of reacted material and the material's half-life to be determined.

In addition, the same data as shown in Figure S11G-I was plotted as natural logarithm and the inverse of the conductivity, together with the trends from extinction spectroscopy. We note that the data can again be fitted to a linear trend for both plots, while better agreement is found for the natural logarithm, in line with a first order rate law. However, as no ambiguous trend is evident from the extracted trends, the data is considered inconclusive with respect to the rate law kinetics and therefore not shown here.

While no clear conclusion can be drawn on the decomposition mechanism from the data presented above, it is clear that the nanomaterial is sensitive to ambient conditions as implied by time dependent extinction and I/V measurements. The measurements ambiguously show that inert exfoliation is required to preserve inherent material characteristics.

7 Comparison to Previous Reports

We note that the work presented in this report is not the only example of liquid exfoliation of metal diborides. To provide a comparison, we have compiled data from previously reported results on the exfoliation of metal diborides, as shown in the table below (Table S1). In summary, the main difference between our results and the existing literature is that we present a method to synthesize metal diboride nanosheets that retain their inherent material properties with only minimal oxidation.

As indicated in the table below, nanomaterials produced in previous studies are chemically modified either intentionally,[21-25] resulting in metal-deficient and functionalized boron-nanosheets, or unintentionally[26-30] due to side-reactions during the process. While these chemically modified nanomaterials may be useful for specific applications, such as flame retardants as suggested by Das and Jasuja,[25] our work focuses on a straightforward method to produce metal diboride nanosheets that maintain their intrinsic properties. This distinction is crucial for applications requiring high electrical conductivity and stability.

To this end, we intentionally did not aim to produce monolayered nanosheets, as this would require extended processing times and additional centrifugation steps, leading to a higher effective surface area which might compromise stability. Our approach emphasizes efficiency and practicality in the production process.

We also note that our work is the first to demonstrate electrically conductive metal diboride nanosheets, highlighting their potential for applications in electronics and other fields where conductivity is essential.

Table S1: *Comparison of previously reported results on the exfoliation of metal diborides in a liquid environment.*

Author	Year	Materials	Method	Liquid Medium	Size/ Thickness*	Chemical modification**	Comments
Das et al.[21]	2015	MgB_2	LPE	H_2O	L > 1 µm<br>T ≈ 3-8 nm	Oxidation observed	Chemically modified MgB_2
Nishino et al.[23]	2017	MgB_2	LPE	H_2O	L ≈ 100 µm<br>T < 10 nm	Oxidation observed	Functionalised Boron-sheets
James, and Jasuja[22]	2017	MgB_2, AlB_2	CE	H_2O + Na_2EDTA	L > 1 µm<br>T ≈ 1-1.7 nm	Oxidation observed	Chelation assisted exfoliation to obtain metal-deficient nanosheets
Ratnam et al.[24]	2017	MgB_2	CE	Ionic Liquid	L > 1 µm<br>T ≈ 2 nm	Oxidation observed	Ion exchange by sonication in ionic liquid
Das and Jasuja[25]	2018	MgB_2	CE	H_2O + TMAOH	L ≈ 1 µm<br>T ≈ 6-20 nm	Oxidation observed	Nanocomposites for flame retardant application
Gunda et al.[26]	2018	MgB_2	LPE	H_2O	L ≈ 40-100nm<br>T ≈ 1-12 nm	Oxidation observed	Recrystallization of MgB_2
John and Anappara[31]	2019	TiB_2	LPE	H_2O	L ≈ 0.2-1µm<br>T ≈ 6-10 nm	Oxidation observed	Luminescent TiB_2 nanosheeets
Patidar et al.[27]	2020	TiB_2	LPE	IPA / H_2O mixtures	L ≈ 0.3-2 µm<br>T ≈ 5-10 nm	Oxidation observed	Co-solvent exfoliation
Gilliam et al.[28]	2021	MgB_2, CrB_2	LPE	30 different solvents	L ≈ 0.05-2 µm<br>T ≈ 1-100 nm	Not studied, mentioned in text	Exfoliability in different solvents
Yousaf et al.[29]	2021	MgB_2, HfB_2, CrB_2, TiB_2, TaB_2, ZrB_2, NbB_2, AlB_2	LPE	ACN, ACT, EtOH, DMF, IPA,	L ≈ 0.02-2 µm<br>T ≈ 1-70 nm	Oxidation observed	PVA composites for mechanical testing
Jiang et al.[32]	2023	MgB_2	CE	H_2O + AcOH	L > 1 µm<br>T ≈ 80 nm	Oxidation observed	MgB_2 nanosheets as solid fuels
Padhi et al.[30]	2023	MgB_2	LPE	EtOH	L ≈ 0.2-0.9µm<br>T ≈ 7-21 nm	Not studied	MgB_2 nanosheets by probe sonication
Jasuja et al.[33]	2023	TiB_2	LPE	H_2O + surfactant	L ≈ 0.1-0.5 µm<br>T ≈ 2-25 nm	Oxidation observed	N_2 absorption on defect sites
This work	2024	MgB_2, CrB_2, ZrB_2	LPE	IPA	L ≈ 0.01-2 µm<br>T ≈ 1-200 nm	Oxidation kinetics studied and minimised	Inert exfoliation and electrical characterization of deposited films

*Sizes and thicknesses have been taken from the individual reports and have been estimated basted on the examples provided in the paper.

**Chemical modification in this context is mainly focused on changes to the spectroscopic response of the nanomaterial compared to the starting material using the data provided in the corresponding report.

8 References

(1) Backes, C.; Szydłowska, B. M.; Harvey, A.; Yuan, S.; Vega-Mayoral, V.; Davies, B. R.; Zhao, P.-l.; Hanlon, D.; Santos, E. J. G.; Katsnelson, M. I.; et al. Production of Highly Monolayer Enriched Dispersions of Liquid-Exfoliated Nanosheets by Liquid Cascade Centrifugation. *ACS Nano* **2016**, *10* 1589-1601.
(2) Ueberricke, L.; Coleman, J. N.; Backes, C. Robustness of Size Selection and Spectroscopic Size, Thickness and Monolayer Metrics of Liquid-Exfoliated WS2. *physica status solidi (b)* **2017**, *254*, 1700443.
(3) Backes, C.; Campi, D.; Szydlowska, B. M.; Synnatschke, K.; Ojala, E.; Rashvand, F.; Harvey, A.; Griffin, A.; Sofer, Z.; Marzari, N.; et al. Equipartition of Energy Defines the Size–Thickness Relationship in Liquid-Exfoliated Nanosheets. *ACS Nano* **2019**, *13*, 7050-7061.
(4) Kelly, A. G.; Hallam, T.; Backes, C.; Harvey, A.; Esmaeily, A. S.; Godwin, I.; Coelho, J.; Nicolosi, V.; Lauth, J.; Kulkarni, A.; et al. All-printed thin-film transistors from networks of liquid-exfoliated nanosheets. *Science* **2017**, *356*, 69.
(5) Ridings, C.; Warr, G. G.; Andersson, G. G. Composition of the outermost layer and concentration depth profiles of ammonium nitrate ionic liquid surfaces. *Phys. Chem. Chem. Phys.* **2012**, *14*, 16088-16095.
(6) Nemes-Incze, P.; Osváth, Z.; Kamarás, K.; Biró, L. P. Anomalies in thickness measurements of graphene and few layer graphite crystals by tapping mode atomic force microscopy. *Carbon* **2008**, *46*, 1435-1442.
(7) Nagashio, K.; Yamashita, T.; Nishimura, T.; Kita, K.; Toriumi, A. Electrical transport properties of graphene on SiO2 with specific surface structures. *J. Appl. Phys.* **2011**, *110*, 024513.
(8) Szendrei, K.; Ganter, P.; Sànchez-Sobrado, O.; Eger, R.; Kuhn, A.; Lotsch, B. V. Touchless Optical Finger Motion Tracking Based on 2D Nanosheets with Giant Moisture Responsiveness. *Adv. Mater.* **2015**, *27*, 6341-6348.
(9) Gosch, J.; Synnatschke, K.; Stock, N.; Backes, C. Comparative study of sonication-assisted liquid phase exfoliation of six layered coordination polymers. *Chem. Commun.* **2023**, *59*, 55-58.
(10) Synnatschke, K.; Shao, S.; van Dinter, J.; Hofstetter, Y. J.; Kelly, D. J.; Grieger, S.; Haigh, S. J.; Vaynzof, Y.; Bensch, W.; Backes, C. Liquid exfoliation of Ni2P2S6: Structural characterisation, size-dependent properties and degradation. *Chem. Mater.* **2019**, *31*, 9127–9139.
(11) Synnatschke, K.; Jonak, M.; Storm, A.; Laha, S.; Köster, J.; Petry, J.; Ott, S.; Szydłowska, B.; Duesberg, G. S.; Kaiser, U.; et al. Sonication-assisted liquid exfoliation and size-dependent properties of magnetic two-dimensional α-RuCl3. *Journal of Physics D: Applied Physics* **2023**, *56*, 274001.
(12) Synnatschke, K.; Moses Badlyan, N.; Wrzesińska, A.; Lozano Onrubia, G.; Hansen, A. L.; Wolff, S.; Tornatzky, H.; Bensch, W.; Vaynzof, Y.; Maultzsch, J.; Backes, C. Sonication-assisted liquid phase exfoliation of two-dimensional CrTe3 under inert conditions. *Ultrasonics Sonochemistry* **2023**, *98*, 106528.
(13) Chacham, H.; Santos, J. C. C.; Pacheco, F. G.; Silva, D. L.; Martins, R. M.; Del'Boccio, J. P.; Soares, E. M.; Altoé, R.; Furtado, C. A.; Plentz, F.; et al. Controlling the Morphology of Nanoflakes Obtained by Liquid-Phase Exfoliation: Implications for the Mass Production of 2D Materials. *ACS Applied Nano Materials* **2020**, *3*, 12095-12105.
(14) Santos, J. C. C.; Prado, M. C.; Morais, H. L. O.; Sousa, S. M.; Silva-Pinto, E.; Cançado, L. G.; Neves, B. R. A. Topological vectors as a fingerprinting system for 2D-material flake distributions. *npj 2D Materials and Applications* **2021**, *5*, 51.

(15) Synnatschke, K. Liquid phase exfoliation and size dependent properties of van der Waals crystals. Dissertation, Heidelberg University, 2021.
(16) Szydłowska, B. M.; Hartwig, O.; Tywoniuk, B.; Hartman, T.; Stimpel-Lindner, T.; Sofer, Z.; McEvoy, N.; Duesberg, G. S.; Backes, C. Spectroscopic thickness and quality metrics for PtSe2 layers produced by top-down and bottom-up techniques. *2D Materials* **2020**, *7*, 045027.
(17) Harvey, A.; Backes, C.; Boland, J. B.; He, X.; Griffin, A.; Szydlowska, B.; Gabbett, C.; Donegan, J. F.; Coleman, J. N. Non-resonant light scattering in dispersions of 2D nanosheets. *Nature Communications* **2018**, *9*, 4553-4564.
(18) McAteer, D.; Godwin, I. J.; Ling, Z.; Harvey, A.; He, L.; Boland, C. S.; Vega-Mayoral, V.; Szydłowska, B.; Rovetta, A. A.; Backes, C.; et al. Liquid Exfoliated Co(OH)2 Nanosheets as Low-Cost, Yet High-Performance, Catalysts for the Oxygen Evolution Reaction. *Advanced Energy Materials* **2018**, *8*, 1702965.
(19) Gabbett, C.; Kelly, A. G.; Coleman, E.; Doolan, L.; Carey, T.; Synnatschke, K.; Liu, S.; Dawson, A.; O'Suilleabhain, D.; Munuera, J.; et al. Understanding how junction resistances impact the conduction mechanism in nano-networks. *Nature Communications* **2024**, *15*, 4517.
(20) Finn, D. J.; Lotya, M.; Cunningham, G.; Smith, R. J.; McCloskey, D.; Donegan, J. F.; Coleman, J. N. Inkjet deposition of liquid-exfoliated graphene and MoS2 nanosheets for printed device applications. *J. Mater. Chem. C* **2014**, *2*, 925-932.
(21) Das, S. K.; Bedar, A.; Kannan, A.; Jasuja, K. Aqueous dispersions of few-layer-thick chemically modified magnesium diboride nanosheets by ultrasonication assisted exfoliation. *Sci. Rep.* **2015**, *5*, 10522.
(22) James, A. L.; Jasuja, K. Chelation assisted exfoliation of layered borides towards synthesizing boron based nanosheets. *RSC Advances* **2017**, *7*, 1905-1914.
(23) Nishino, H.; Fujita, T.; Yamamoto, A.; Fujimori, T.; Fujino, A.; Ito, S.-i.; Nakamura, J.; Hosono, H.; Kondo, T. Formation Mechanism of Boron-Based Nanosheet through the Reaction of MgB2 with Water. *The Journal of Physical Chemistry C* **2017**, *121*, 10587-10593.
(24) Ratnam, D.; Das, S. K.; Jasuja, K. Ionic Liquid Assisted Exfoliation of Layered Magnesium Diboride. *IOP Conference Series: Materials Science and Engineering* **2017**, *225*, 012111.
(25) Das, S. K.; Jasuja, K. Chemical Exfoliation of Layered Magnesium Diboride To Yield Functionalized Nanosheets and Nanoaccordions for Potential Flame Retardant Applications. *ACS Appl. Nano Mater.* **2018**, *1*, 1612-1622.
(26) Gunda, H.; Das, S. K.; Jasuja, K. Simple, Green, and High-Yield Production of Boron-Based Nanostructures with Diverse Morphologies by Dissolution and Recrystallization of Layered Magnesium Diboride Crystals in Water. *ChemPhysChem* **2018**, *19*, 880-891.
(27) Patidar, R.; Gunda, H.; Varma, A. K.; Gawas, R.; Das, S. K.; Jasuja, K. Co-solvent exfoliation of layered titanium diboride into few-layer-thick nanosheets. *Ceram. Int.* **2020**, *46*, 28324-28331.
(28) Gilliam, M. S.; Yousaf, A.; Guo, Y.; Li, D. O.; Momenah, A.; Wang, Q. H.; Green, A. A. Evaluating the Exfoliation Efficiency of Quasi-2D Metal Diboride Nanosheets Using Hansen Solubility Parameters. *Langmuir* **2021**, *37*, 1194-1205.
(29) Yousaf, A.; Gilliam, M. S.; Chang, S. L. Y.; Augustin, M.; Guo, Y.; Tahir, F.; Wang, M.; Schwindt, A.; Chu, X. S.; Li, D. O.; et al. Exfoliation of Quasi-Two-Dimensional Nanosheets of Metal Diborides. *J. Phys. Chem. C* **2021**, *125*, 6787-6799.
(30) Padhi, S. K.; Liu, X.; Valsania, M. C.; Andreo, L.; Agostino, A.; Alessio, A.; Pastero, L.; Giordana, A.; Wu, Z.; Cravotto, G.; Truccato, M. Structure and physicochemical

properties of MgB_2 nanosheets obtained via sonochemical liquid phase exfoliation. *Nano-Structures & Nano-Objects* **2023**, *35*, 101016.
(31) John, S. K.; Anappara, A. A. Aqueous dispersions of highly luminescent boron-rich nanosheets by the exfoliation of polycrystalline titanium diboride. *New Journal of Chemistry* **2019**, *43*, 9953-9960.
(32) Jiang, Y.; Ka, D.; Huynh, A. H.; Baek, J.; Ning, R.; Yu, S.-J.; Zheng, X. Exfoliated Magnesium Diboride (MgB_2) Nanosheets as Solid Fuels. *Nano Lett.* **2023**, *23*, 7968-7974.
(33) Rasyotra, A.; Thakur, A.; Mandalia, R.; Ranganathan, R.; Jasuja, K. Nitrogen adsorption via charge transfer on vacancies created during surfactant assisted exfoliation of TiB_2. *Nanoscale* **2023**, *15*, 8204-8216.
